# Adaptation of hippocampal spatial and contextual representations to task structure

**DOI:** 10.1126/sciadv.adu4899

**Published:** 2025-11-14

**Authors:** Rita Nyilas, Atilla B. Kelemen, Balázs Lükő, Máté Sümegi, Balázs B. Ujfalussy, Judit K. Makara

**Affiliations:** ^1^ Laboratory of Neuronal Signaling, HUN-REN Institute of Experimental Medicine, Budapest 1083, Hungary.; ^2^ Laboratory of Biological Computation, HUN-REN Institute of Experimental Medicine, Budapest 1083, Hungary.; ^3^ Laboratory of Cellular Neurophysiology, HUN-REN Institute of Experimental Medicine, Budapest 1083, Hungary.

## Abstract

The hippocampus is fundamental for spatial and contextual memory. CA1 pyramidal neurons (CA1PNs) exhibit spatially tuned activity during navigation, but how their context-dependent tuning evolves during learning and upon context change is incompletely understood. We monitored Ca^2+^ activity of dorsal CA1PNs in mice in a virtual go/no-go task series that required both spatial navigation and rule learning based on nonspatial environmental features. Spatial and context-dependent activity of CA1PNs developed sequentially during learning, parallel with behavioral performance. Upon switching to a new task condition involving familiar and novel environments, neuronal activity disorganized rapidly and extensively even in the unchanged environment. In contrast, reward reversal in unchanged environments induced more gradual activity changes, associated with behavioral adaptation. Our results indicate that, during learning a spatial-contextual task, CA1PNs initially generalize between environments coding primarily spatial position, but, with experience, their activity gradually reorganizes to form partially distinct representations within a shared contextual framework.

## Teaser

Hippocampal neuronal maps are actively restructured during learning and upon context change to represent task structure.

## INTRODUCTION

Performing different tasks in similar environments, like playing football or basketball in the same field or playing checkers or chess on the same board, requires flexible representations. To achieve this, the brain has to learn to encode the features of the environment relevant for the current task (where is the goal?) as well as task rules (can you touch the ball with your hands?). Developing efficient task-specific representations is thought to be crucial for mastering different games (*1*, *2*) and solving context-specific problems in general, but how it is accomplished by the brain is not well understood.

The hippocampus has been implicated in flexibly encoding environmental variables including spatial location (*3*, *4*), objects (*5*, *6*), conspecifics (*7*), nonspatial sensory cues (*8*, *9*), or other, more abstract contextual features (*10*–*12*). In particular, a large fraction of PNs of the rodent hippocampus shows position-dependent activity (i.e., a place field) during exploration (*4*, *13*). Position tuning of the cells appears quickly, in most cases within the first few minutes of exploration (*14*–*16*) even in virtual environments (*17*, *18*), and different locations are usually assigned to an orthogonal neural code (*19*–*21*), suggesting that the hippocampus provides unique state representations that can be used for rule learning.

Beyond their well-recognized spatial tuning, the activity of hippocampal PNs has also been shown to be influenced by nonspatial and contextual features associated with environments (*22*–*24*). Context includes the combination of constant sensory features of the surroundings as well as nonsensory and/or internal variables such as task structure or demand, which can vary in time even in the same environment (*10*, *25*–*33*). Identifying the task-relevant environmental features among the multitude of irrelevant stimuli is a hard problem that needs time and experience (*2*). Indeed, hippocampal representations of similar environments have been shown to diverge gradually with extended experience (*34*–*37*), indicating that the neuronal code incorporates greater details with further experience (*38*). Similarly, neuronal activity reflecting the acquisition of abstract task rules (e.g., splitter cells in a delayed alternation task) appears later during learning than activity reflecting available sensory input (e.g., place cells) (*39*, *40*). How learning a particular task drives selection of relevant features (such as location, sensory cues or valence, and the relational rules between them) and their integration into hippocampal representations are not yet well elucidated.

In addition to the gradual refinement of the hippocampal state representation during context learning, sudden changes in sensory cues, introduction of salient stimuli, or changing the location of reward or the behavioral strategy to obtain it can induce rapid remapping in hippocampal place cells (*10*, *27*–*32*, *41*–*44*). How a sudden context change reorganizes the hippocampal code and how it interacts with the slower process of gradual refinement of the representations of similar environments are poorly understood. In particular, in complex situations where related environments are associated with different tasks, changing the task in one environment can influence the representation of the other, similar environment, a strong evidence for hierarchical task representation (*45*, *46*). Conversely, certain features of the representation could be preserved across major changes in task or context (*47*), indicating that the hippocampal code for place and events could be factorized (modular or compositional).

Here, we set out to study the emergence of spatial and context-selective CA1PN representations during learning a complex task composed of multiple environments and to investigate the dynamic changes of neuronal activity on the single-cell and population levels upon alterations in sensory or nonsensory contextual information. To this end, we developed a virtual goal-directed go/no-go task series that required both spatial navigation and rule learning based on nonspatial features of environments, and we used two-photon (2P) Ca^2+^ imaging to chronically monitor the activity of large populations of dorsal CA1PNs.

## RESULTS

### Behavior and in vivo Ca^2+^ imaging in a virtual go/no-go task

**Fig. 1.**
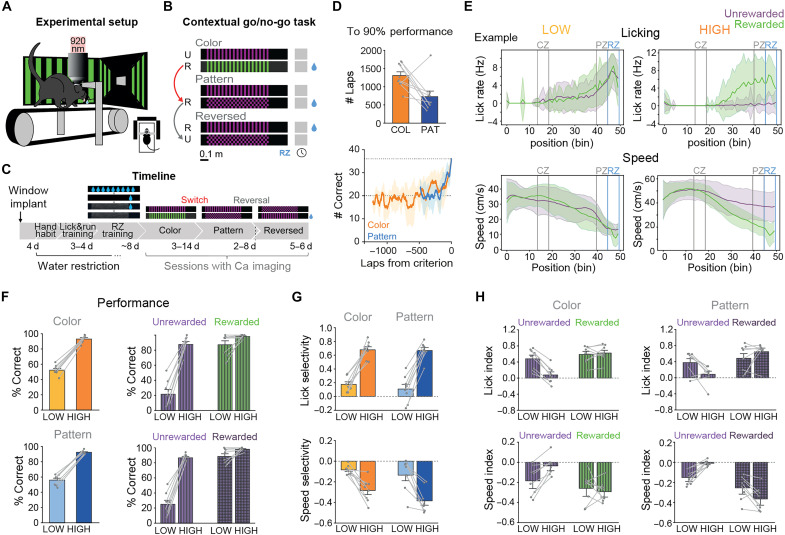
Mice learn to discriminate between two visually distinct corridors in a contextual go/no-go task. (**A**) Behavioral setup. Head-fixed mice navigated on a cueless belt in a virtual reality environment projected by computer monitors around the mice (bottom right inset: schematic top view). (**B**) Corridor pairs used in the three task conditions. Water reward was delivered in only one corridor (drop) operantly in an uncued reward zone (RZ) located near the end of the corridors. Rewarded (R) and unrewarded (U) corridors were presented in a pseudorandom order. Corridors were separated by gray screens between laps, with longer duration after incorrect choice. (**C**) Experimental timeline (see Materials and Methods). Hand habit, hand habituation. d, days. (**D**) Top: Number of laps needed to achieve 36 of 40 correct choices in the color (COL; *n* = 10 mice) and in the pattern (PAT; *n* = 9 mice) task. Bottom: Number of correct laps (moving average from 40 laps) across animals (means ± SD of 5+ mice) in the Color and Pattern task aligned to the first reach of the criterion. (**E**) Example mouse behavior in LOW- (left) and HIGH-performance (right) sessions in the Color condition. Top: Lick rate per track position in R (green) and U (purple) corridors. Bottom: Running speed. CZ, control zone; PZ, prereward zone. Data show means (line) ± SD (shading) across laps. (**F**) Performance in LOW versus HIGH sessions in the Color (top) and Pattern (bottom) tasks (*n* = 8 mice with imaging). (**G**) Intercorridor lick and speed selectivity (see Materials and Methods) between U versus R corridors. (**H**) Intracorridor lick and speed index calculated separately in U and R corridors (see Materials and Methods). In [(D), top] and (F) to (H), connected dots correspond to individual mice, and bars show means ± SE across mice. Statistical analysis results corresponding to the figure data: table S1.

Water-restricted, head-fixed Thy1-GCaMP6s-expressing transgenic mice were trained in virtual reality (Fig. 1A) to navigate in and discriminate between two visually distinct, pseudo-randomly presented corridors based on either their color (Color condition) or their pattern (Pattern and Reversed conditions; Fig. 1, B and C). In the first part of the task (Color condition), the mice learned to run along a 106-cm-long corridor with either green or purple striped wall pattern, followed by a cueless black corridor segment containing a hidden reward zone (RZ) where the animals had to lick to receive water in the rewarded (R) corridor (green striped) but withhold licking in the unrewarded (U) corridor (purple striped). Completed laps were followed by a cueless gray screen (gray zone), with longer duration in incorrect laps for negative reinforcement. Running speed and lick rate were monitored as behavioral readouts, and performance was assessed by the ratio of correct laps. Once the mice learned the task in the Color condition (expert: ≥90% correct in 40 laps), they progressed to the Pattern condition where they had to discriminate corridors based on their wall patterns [checked (R) or striped (U), both purple]. The second task condition was generally acquired faster than the first one (Fig. 1D). Last, after the mice became experts in the Pattern condition, we swapped the reward between the corridors [Reversal, checked (U) or striped (R), both purple; Fig. 1, B and C] and followed learning in the Reversed condition.

We first characterized the behavioral correlates of learning. In both the Color and Pattern conditions, at the early stages of familiarizing with the corridors and the task, lick rate increased and running speed decreased similarly as mice ran along the two corridors toward the RZ (Fig. 1E), and performance was at chance level (Fig. 1F; LOW, fraction of correct laps 52.35 ± 2.21% and 56.04 ± 2.41% (means ± SE) in Color and Pattern, respectively), indicating that the mice have learnt the location of the RZ but have not yet understood the task rule. During the course of learning, mice increasingly slowed down and licked before the RZ in the R corridor but withheld licking in the U corridor with little change in velocity (Fig. 1E, HIGH), as indicated by an increase of intercorridor lick and speed selectivities (Fig. 1G) and changes in intracorridor lick and speed indices (Fig. 1H). The behavioral changes took place predominantly in U, but an increase in performance was also observed in R, although to a smaller extent. These behavioral changes altogether indicated early learning of the spatial structure of the task but slower and gradual understanding of the reward contingencies, in both the Color and Pattern conditions. Similar sequence of behavioral changes were observed in the Reversed condition as well (fig. S1). On the basis of the overall behavioral performance in the corridor pairs, we took one low-performance (LOW, fraction of correct laps < 0.65) and one high-performance (HIGH, fraction of correct laps > 0.84; Fig. 1F) epoch of imaging sessions from eight animals and used them for further analysis [see fig. S3 for data acquired during intermediate-performance (MED) imaging epochs].

**Fig. 2.**
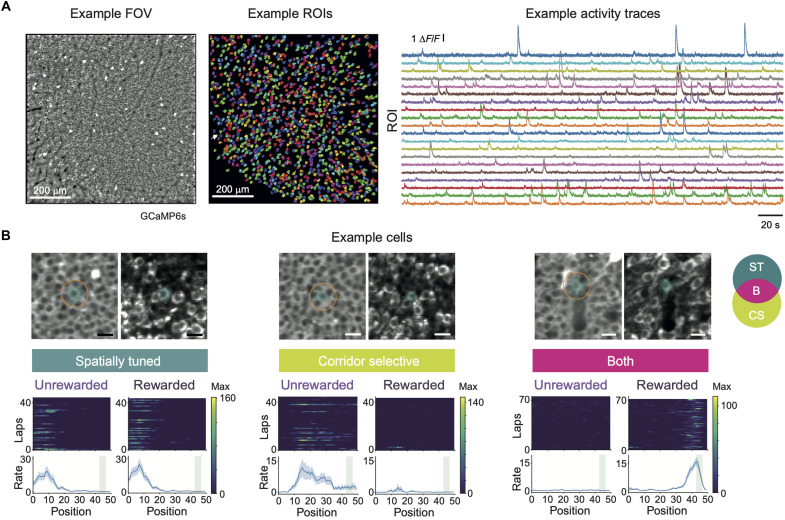
Task-related activity of CA1PNs recorded by 2P Ca^2+^ imaging. (**A**) Left: Example imaging field of view (FOV; mean enhanced Suite2p image at 920 nm). Middle: Segmented regions of interest (ROIs) of the same FOV (Suite2p image, see Materials and Methods). Right: Extracted Δ*F*/*F* Ca^2+^ traces from selected ROIs in the middle panel. (**B**) Examples for spatially tuned (ST; left), corridor-selective (CS; middle), and both spatially tuned and corridor-selective [(B); right] cells (see Materials and Methods) recorded in Pattern condition. Top row: 2P images of the cells (left, mean enhanced image; right, maximum intensity projection). Selected cells are indicated by orange circles, and the ROI masks are shown in cyan. Bottom panels: Lap-by-lap activity and average activity (means ± SE, line and shading) per position bins along the U and R corridors. Color codes range from 0 to maximum activity (inferred spike rate).

To monitor the activity of large populations of hippocampal CA1PNs during the evolution of task-relevant behavioral responses, we used 2P imaging through an implanted hippocampal imaging window and recorded GCaMP6s-reported Ca^2+^ signals in up to ~1500 cells simultaneously in each mouse (Fig. 2A). We computed several metrics (spatial reliability, spatial tuning specificity, and spatial information content) to determine spatial tuning in both corridors and determined corridor-specific differences in activity (corridor selectivity) for each neuron using bootstrapping (see Materials and Methods and fig. S2A). On the basis of this analysis, we identified (i) spatially tuned cells and (ii) corridor-selective cells (Fig. 2B and fig. S2B; note that individual cells may belong to both groups) and explored how neuronal coding of position and corridor evolved in parallel with behavioral adaptation during learning.

### Development of spatial tuning and corridor selectivity in CA1PNs during learning

**Fig. 3.**
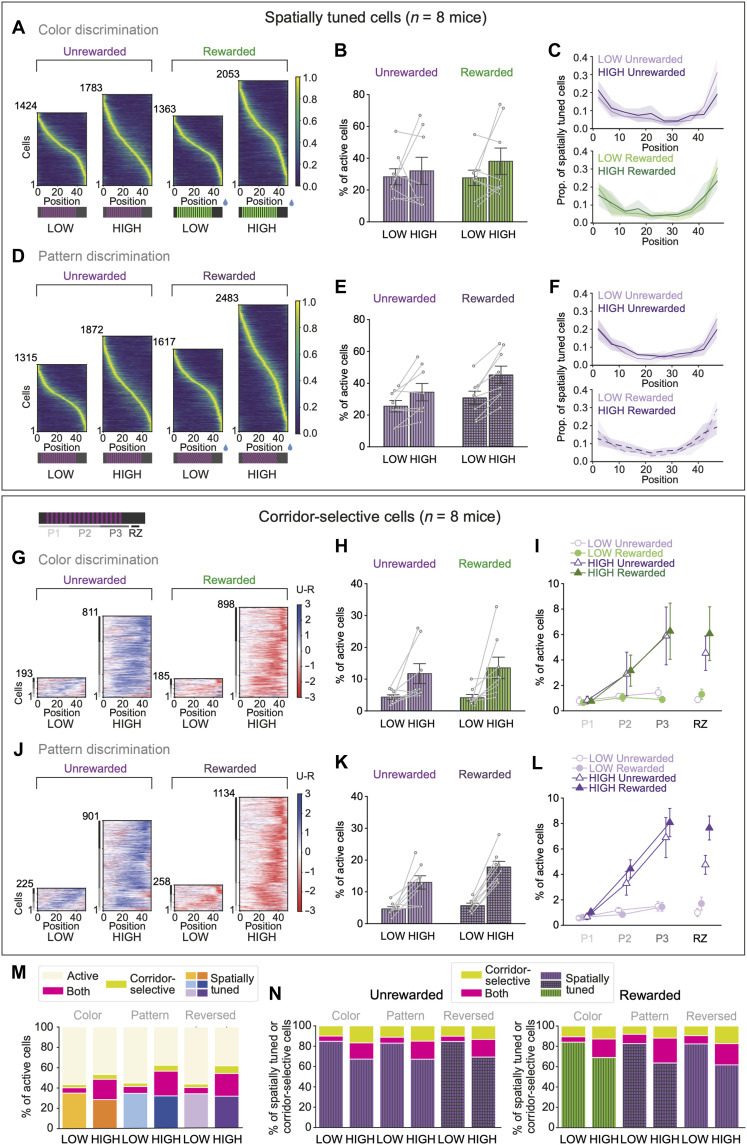
Development of spatial tuning and context-encoding by CA1PNs with learning. (**A** and **D**) Color-coded normalized population activity rate maps of CA1PNs with significant spatially tuned activity in Color (A) and Pattern (D) condition in LOW- and HIGH-performance sessions. Cells were pooled across sessions recorded from 8 mice and were sorted by the location of peak activity. (**B** and **E**) Percent of spatially tuned cells within all active cells in the two corridors in the Color (B) and the Pattern (E) condition. (**C** and **F**) Proportion of spatially tuned cells along 50 position bins of U and R corridors in LOW and HIGH sessions (means ± SD, *n* = 8 mice). (**G** and **J**) Normalized activity difference of the neurons selective for the Unrewarded (left) and Rewarded (right) corridors. In each plot, neurons are ordered by the location zone of their selectivity (P1 to P3, patterned zones 1 to 3; RZ, reward zone; shades of gray on the left, see inset on the top). Neurons selective in multiple zones are shown in each. Cells were pooled across eight sessions from eight mice. (**H** and **K**) as in (B) and (E), for corridor-selective cells. (**I** and **L**) Percent of zone selective cells (significantly selective in the indicated zone) of the U or R corridor in LOW and HIGH sessions of (I) Color or (L) Pattern condition (means ± SD, *n* = 8 mice). (**M**) Percent of spatially tuned only (orange, blue, or purple per condition), corridor-selective only (light green), and both corridor-selective and spatially tuned (magenta) cells within all active cells in LOW and HIGH sessions (Color, *n* = 8; Pattern, *n* = 8; Reversed, *n* = 5 mice). (**N**) Same as (M) within all tuned or selective cells in the different corridors. Statistical analysis: table S1.

To identify the changes of spatial and context-related CA1PN Ca^2+^-activity associated with learning, we first compared the fraction of spatially tuned and corridor-selective cells in LOW versus HIGH sessions (Fig. 3) within active cells (≥1 Ca^2+^ transient per minute). The fraction of active cells within all ROIs was stable between LOW versus HIGH sessions in both conditions (Color, *P* = 0.161; and Pattern, *P* = 0.575; Wilcoxon test).

We observed a sizable fraction of spatially tuned neurons already in LOW sessions, i.e., at the early stages of learning the task (Fig. 3, A, B, D, and E). Spatially tuned cells tiled the entire track both in the U and R corridors, with the start and the RZ markedly overrepresented (Fig. 3, A, D, C, and F). Compared to the LOW sessions, the ratio of spatially tuned cells moderately increased in HIGH sessions, particularly in the R corridor (Fig. 3, B and E).

Corridor-selective cells were also present at the beginning of learning, albeit in a much lower proportion than spatially tuned cells (Fig. 3, G, H, J, and K). Corridor-selective cells covered relatively evenly the entire length of corridors (Fig. 3, G and J). The initially low fraction of corridor-selective cells increased markedly with experience in both the R and U corridors in both the Color and Pattern conditions (Fig. 3, H and K; see also fig. S3 for intermediate-performance sessions). This change was location dependent, being the most pronounced in and before the RZ, especially in the R corridor (Fig. 3, I and L). These results were similar in all conditions (Fig. 3, see fig. S4 for the Reversed condition).

As spatial tuning and corridor selectivity are not mutually exclusive features of neurons, we calculated the overlap and relative ratios of spatially tuned and corridor-selective cells in LOW and HIGH sessions in the three task conditions (Fig. 3, M and N; for intermediate sessions in Color and Pattern, see fig. S3). The ratio of cells that were both spatially tuned and corridor selective strongly increased with learning, in part at the expense of the only spatially tuned neurons (Fig. 3, M and N; for intermediate sessions in Color and Pattern, see fig. S3), whereas the ratio of spatial-only cells decreased and the ratio of corridor-selective-only cells did not change over the course of learning (Fig. 3M). Furthermore, at the beginning of learning in the subsequent condition, the cell proportions returned to values close to that observed in LOW performance in the previous condition. Notably, these changes were similar in both the R and in the U corridors (Fig. 3N), despite the U corridor remaining completely unchanged when progressing from Color to Pattern condition. Cells with spatially tuned and/or corridor-selective activity were intermingled and showed no obvious bias in their topographic location within the imaged field of view (FOV) (fig. S5).

### Population-level evolution of spatial and contextual representations

**Fig. 4.**
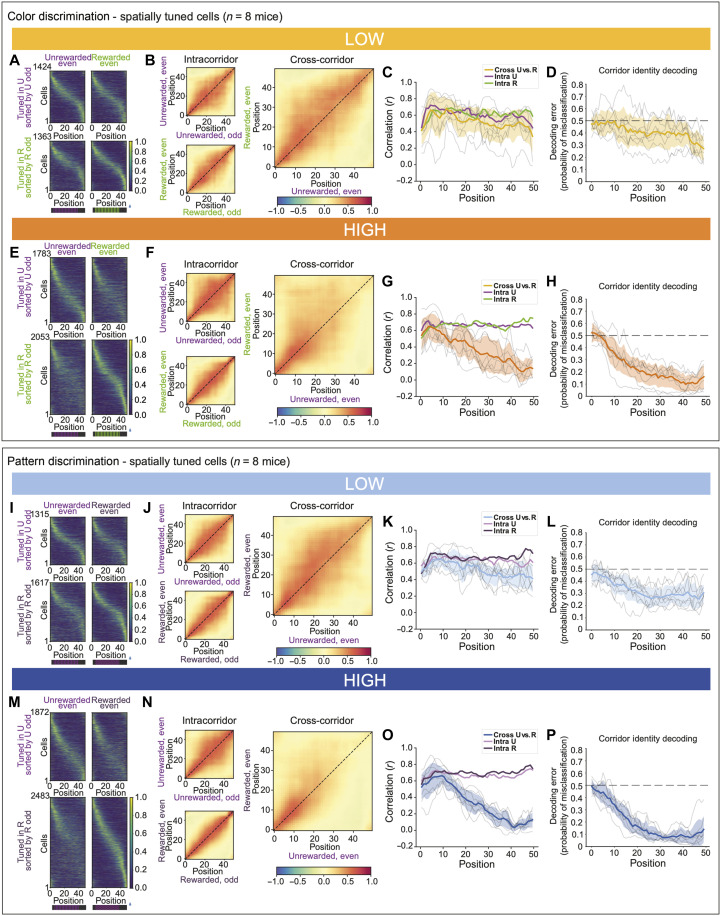
Spatial representations of corridor pairs become less correlated near the RZ with learning. (**A** and **E**) Cross-sorted, color-coded normalized activity rate maps of cells with significant spatially tuned activity in the rewarded (R) and unrewarded (U) corridors in LOW (A) and HIGH (E) sessions. Cells were pooled from eight mice and sorted by the location of peak activity using the odd laps of the corridor indicated. Adjacent rate maps show the same cells in the same order. (**B** and **F**) Left: PV correlation of rate maps calculated from odd versus even laps in the U (top) or R (bottom) corridor. Right: PV correlation of the rate maps in the two corridors in Color condition in LOW (B) and HIGH (F) sessions (average across *n* = 8 mice). (**C** and **G**) The diagonals of the PV-correlation matrices between the U versus R corridors of individual animals (gray, *n* = 8) and their means ± SD (yellow/orange). Correlations were similarly high in the first and last 10 spatial bins in LOW, but decreased along the corridors in HIGH. Purple and green lines represent the mean diagonal of the corresponding intracorridor rate map correlation matrices. (**D** and **H**) Decoding error by Bayesian decoding of corridor identity (color, means ± SD; gray, individual sessions) as a function of position in LOW [(D), yellow] and HIGH [(H), orange] sessions. (**I** to **P**) Qualitatively similar results were observed in Pattern condition as in (A) to (H). Statistical analysis: table S1.

Next, we analyzed how the changes in neuronal activities influenced the population-level representations, by calculating population vector correlation (PV correlation; i.e., correlation of the position-dependent rate maps, see Materials and Methods) of spatially tuned cells in the U and R corridors (Fig. 4).

In LOW sessions, spatially tuned cells showed similar activity patterns in the R and U corridors (Fig. 4, A and I), and, accordingly, the population-level spatial representations of the two corridors were highly correlated along the whole extent of the tracks (Fig. 4, B, C, J, and K). However, in HIGH sessions, the spatial representations of the U and R corridors became increasingly decorrelated from the track start toward the RZ (Fig. 4, F, G, N, and O). Similar results were found in all conditions (see fig. S4 for the Reversed condition).

Next, we trained a Bayesian decoder to predict corridor identity based on active neurons. We found that the corridor identity error was near chance level for the LOW sessions and decreased significantly in HIGH sessions (Fig. 4, D, H, L, and P). The improvement in decoding with learning was the most pronounced at spatial positions close to the RZ (Fig. 4, D, H, L, and P). In contrast, the encoding of the position within the corridors was relatively less affected by the above changes in the corridor representations (fig. S6).

### Complex adaptation of single-neuron tuning during learning

**Fig. 5.**
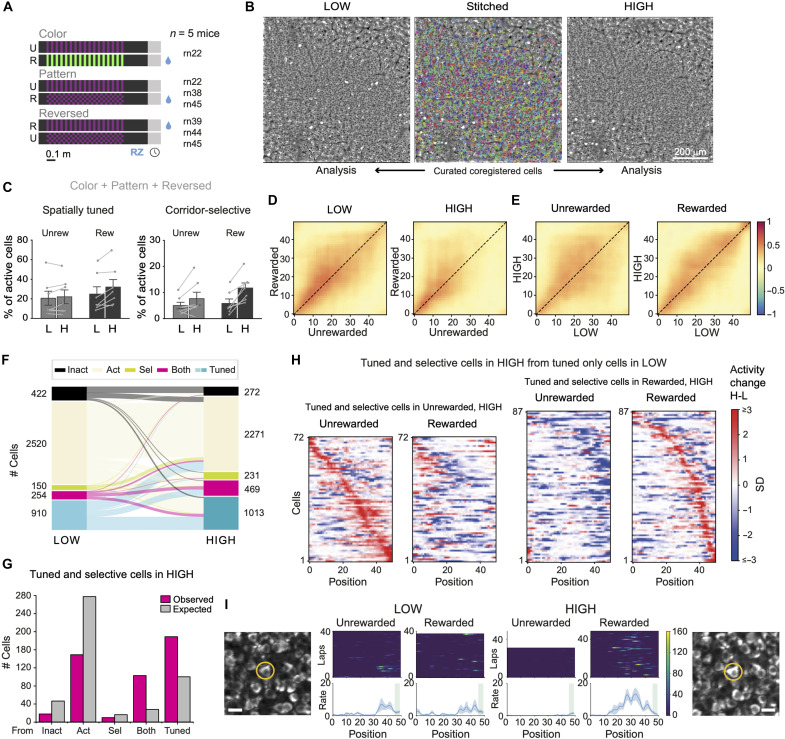
Adaptation of individual CA1PNs during contextual learning. (**A**) Summary of the conditions where individual neurons could be tracked from LOW to HIGH (*n* = 7 epoch pairs in *n* = 5 mice were pooled for the analysis). (**B**) Representative FOV in LOW (left) and HIGH (right). The identified ROIs with stitched activity are colored (middle). (**C**) Percent of spatially tuned (left) and corridor-selective (right) cells within all active coregistered cells in the U and R corridors. Data points, individual mice; bar plots, means ± SE (*n* = 7 sessions). L, LOW; H, HIGH. (**D**) PV correlation between the spatial maps in the U and R corridors at different positions in LOW and HIGH epochs (average across *n* = 7 sessions). (**E**) PV correlation between LOW and HIGH epochs in the U (left) and R (right) corridors (average across *n* = 7 sessions). (**F**) Sankey diagram of the activity transition of CA1PNs during the increase of performance. (**G**) Numbers of cells becoming tuned and selective from the different activity categories (observed, magenta) versus the numbers of cells expected from a random transition process (gray). (**H**) Normalized activity difference between HIGH and LOW epochs for spatially tuned cells selective in U (left) and in R (right), shown in both corridors. The activity difference is normalized separately for each cell by dividing it with the SD across all positions. (**I**) Example CA1PN (2P image on the left and right panels, maximum intensity projection) showing spatially tuned activity in LOW and tuned and corridor-selective activity in HIGH. Statistical analysis: table S1.

In a subset of experiments, the performance of the animal increased from LOW to HIGH either within a single imaging session or in session pairs with the same FOV (on the same days; conditions: *n* = 1 Color, *n* = 3 Pattern, and *n* = 3 Reversed; Fig. 5A), allowing tracking the activity of identified neurons during the learning process (Fig. 5B). In these recordings, the proportion of spatially tuned and corridor-selective cells (Fig. 5C) and the PV correlations in LOW and HIGH (Fig. 5D) changed qualitatively similarly to that described for distinct cell populations. Interestingly, the divergent representation of U and R with learning (Fig. 5D) was accompanied by relatively stable correlation within the same corridors across LOW and HIGH (Fig. 5E), suggesting no drastic reorganization of activity and tuning of individual cells.

To explore this in more detail, we examined the activity changes in individual neurons. Because learning of the tasks was most prominently associated with an increased population of cells that are both corridor selective and spatially tuned (i.e., dually selective), we particularly focused on the coding history of these CA1PNs. We found similar changes across the different conditions, and, therefore, the data were pooled.

First, our analysis showed that the group of cells that are dually selective in HIGH are derived from three major coding groups in LOW: ~22% of cells were already dually selective and retained this property; ~32% of cells were active but nonselective; and ~40% were spatially tuned but not corridor selective (Fig. 5F). Statistical analysis confirmed that these observed proportions were significantly different from that expected from random conversion of cells to become dually selective with equal chance from any of the groups (Fig. 5G). Thus, while the remapping route is not exclusive, the largest fraction of the newly developed dually selective cells arises from cells that were only spatially tuned before.

Second, examining the changes in activity in the two corridors, we found that cells that were dually selective in HIGH generally increased their activity in the corridor that they became selective for, as well as suppressed their activity in the other corridor (Fig. 5, H and I), suggesting that complex plasticity mechanisms underlie their adaptation during the learning process. When we examined the changes in those cells that developed from spatially tuned only to dually selective, we found that the increase in activity often occurred at positions directly preceding the original peak activity (Fig. 5, H and I, and fig. S7).

In summary, parallel with performance increase in the virtual go/no-go task, the fraction of spatially tuned and corridor-selective cells markedly increased primarily near the reward location, and this change took place in both types of corridors and in all experimental conditions. Together, our results suggest that, consistent with the behavioral adaptation during learning the task, hippocampal CA1PNs initially generalize between environments (coding spatial position only), but, with experience, they dynamically reorganize their activity and form partially distinct representations according to the behavioral relevance of a given environment.

### Rapid reorganization of spatial and corridor-selective CA1PN representations upon context switch

**Fig. 6.**
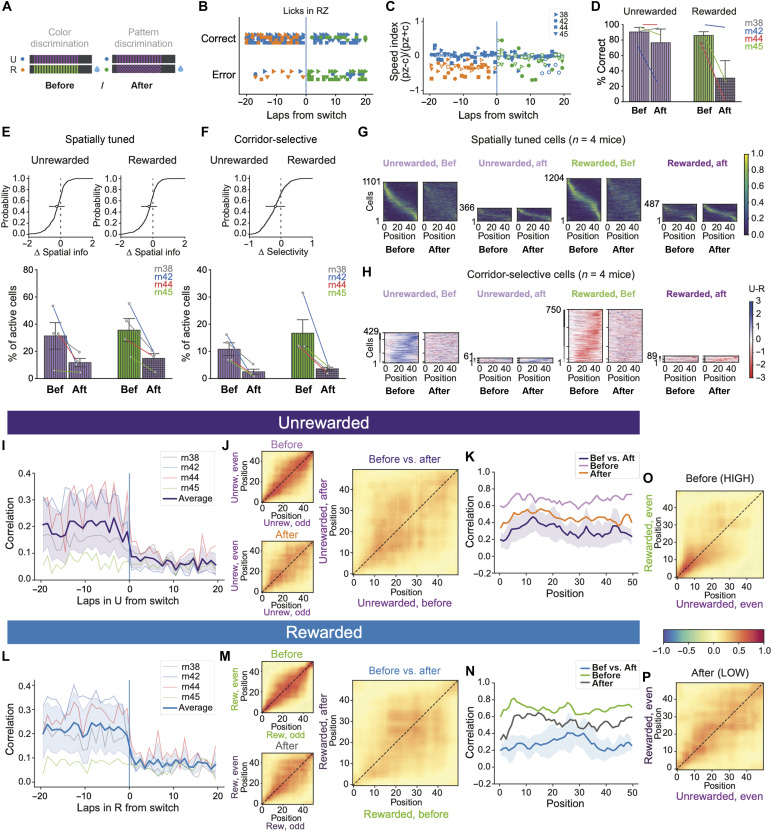
Abrupt reorganization of CA1PN activity upon context switch. (**A**) Schematic of the Switch. (**B** and **C**) Correct and error laps (B) and speed index (C) in *n* = 4 mice (symbols) in 20 laps before and after Switch (colors, corridors as in A). (C) Filled symbols, correct lap; empty symbols, incorrect lap. (**D**) Performance before and after Switch in the U and R corridors. (**E**) Top: Cumulative probability of difference in spatial information before and after Switch in the Unrewarded (*n* = 669 cells, pooled) and Rewarded corridors (*n* = 815 cells). Symbols and whiskers, means ± SD. Bottom: Fraction of spatially tuned cells before and after Switch. (**F**) Top: Cumulative probability of the difference in corridor selectivity before versus after Switch (*n* = 1160 cells). Bottom: Fractions of corridor-selective cells before and after Switch. (**G**) Cross-sorted, normalized activity rate maps of cells exhibiting spatially tuned (G) or corridor-selective (H) activity before or after Switch in the corridors indicated on top. (**H**) Normalized activity difference of corridor-selective neurons before and after Switch. (**I** and **L**) Average lap-to-lap correlation around Switch in the U (I) and R (L) corridors. Thick line and shadow, means ± SD. (**J** and **M**) Left: PV correlation of rate maps calculated from odd versus even laps in the U (J) and R (M) corridors before and after Switch (*n* = 4 mice). Right: PV correlation between the spatial maps before and after Switch. (**K** and **N**) Diagonals of the PV-correlation matrices before versus after Switch in the U (K) and R (N) corridors (dark purple in K and blue in N; with shading, ± SD). Light purple/orange (K) and green/dark gray (N) lines, correlations between even and odd laps before and after Switch. (**O** and **P**) PV correlations of spatial positions between the two corridors before (O) and after (P) Switch. Symbols and whiskers in (D) and [(E) and (F), bottom], means ± SE. Statistical analysis: table S1. Bef, before; Aft, after.

As shown above, changing the task from the Color to the Pattern condition resulted in a marked reset in the characteristics of task-related hippocampal activity patterns (to lower ratios of feature-specific cells and less distinctive representations across corridors). To better understand the dynamics of these changes, in four of the eight mice that were experts in Color, we performed Ca^2+^ imaging during uncued switching to the Pattern condition, allowing the assessment of Switch-induced activity changes in individual neurons. Notably, the Switch involved changing the wall pattern of the R corridor to one that the mice had never experienced before (from green striped to purple checked), but the U corridor (purple striped) remained unchanged (Fig. 6A).

The mice immediately noticed the intrasession Switch upon the first presentation of the novel corridor, and, although they exhibited heterogeneous behaviors in the new condition (Fig. 6, B and C; and fig. S8, A and B), altogether, the Switch resulted in a marked drop in performance (Fig. 6, B and D, fig. S8A) and an increase in speed index in the R corridor (Fig. 6C and fig. S8B).

Irrespective of the individual behavioral differences across mice, the Switch from Color to Pattern induced a strong decrease in the fractions of both the spatially tuned and the corridor-selective cells in all four mice (Fig. 6, E and F): most cells lost their spatial selectivity and their corridor-specific activity after the Switch. Notably, this change occurred not only in the R corridor (which was completely novel) but also in the U corridor that remained identical before and after the Switch (Fig. 6, E and F). Furthermore, the cells that were spatially tuned and/or corridor selective in the Color condition typically did not retain their original tuning after the Switch in any of the corridors (Fig. 6, G and H; see fig. S9A for example cells). In a subset of mice, we repeated the Switch session on the same or the following day after reexposure to the original Color context and found that a large fraction of the cells regained their previous tuning properties in the Color condition and then again remapped upon the repeated Switch to the Pattern condition (fig. S10).

We next analyzed the changes in population-level representations of the corridors by the same CA1PNs recorded during the Switch. The average lap-to-lap correlation of population activity was stable in the last 20 laps before the Switch but exhibited a sharp drop after the Switch in both the R and U corridors (Fig. 6, I and L). The correlation remained low in the first 20 laps after the Switch in both corridors (Fig. 6, I and L), indicating no rapid emergence of reliable new activity maps.

We further examined the impact of Switch-induced activity reorganization on the spatial representations of the corridors. PV correlation between the even and odd laps was high before the Switch in both the U and R corridors, indicating stability of the spatial representations in HIGH sessions (Fig. 6, J, K, M, and N). Spatial representations were less stable after the switch (lower PV correlations than before) and differed significantly from preswitch representations (lowest PV correlations between pre and post) (Fig. 6, J, K, M, and N). Finally, when we compared the spatial correlations between the U versus R corridors before and after the Switch, we found that the decorrelated population activity near the RZ, characteristic to HIGH sessions (Fig. 6O), disappeared after the Switch (Fig. 6P), similarly to the difference presented earlier between the Color HIGH and Pattern LOW conditions for all eight mice (Fig. 4, F and J). Despite the strong reorganization of tuning, the number of active cells did not differ significantly before (650 ± 84, mean ± SE) versus after (710 ± 121) the Switch (*P* = 0.273, Wilcoxon test).

These results demonstrate that CA1PN representations reorganize rapidly, robustly, and persistently upon an abrupt change from a learned to a novel context, and this reorganization extends even to those components of the context that remain unaltered. These findings suggest that, instead of separate representations of the individual environments (corridors), CA1PNs developed a hierarchical task representation with distinct codes formed for the different task components.

### Gradual reorganization of spatial and context-selective CA1PN representations upon reward reversal

**Fig. 7.**
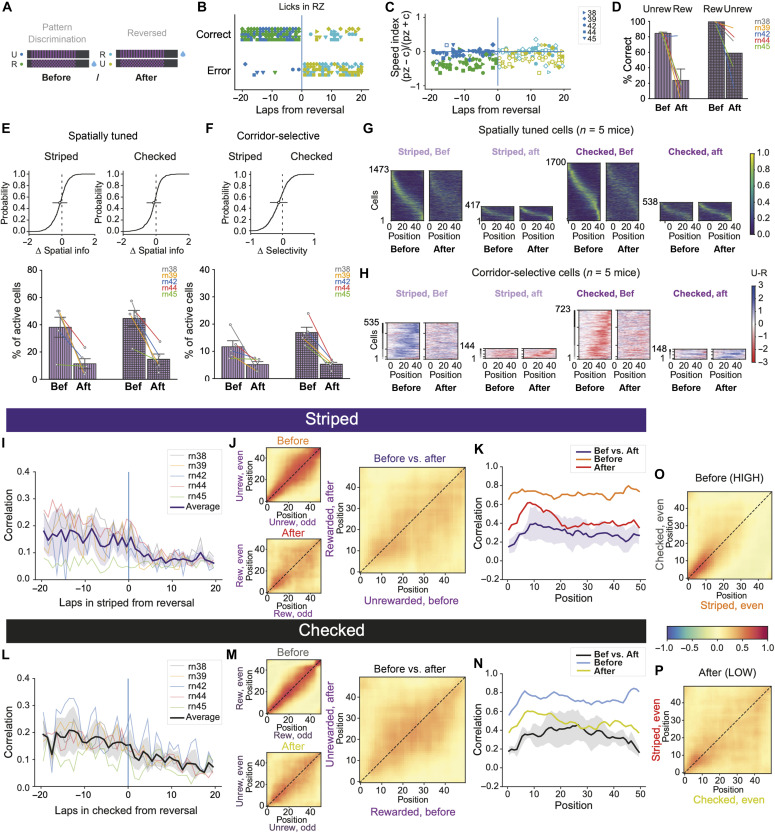
Gradual reorganization of CA1PN activity upon Reversal. (**A**) Schematic of Reversal sessions. (**B** and **C**) Correct and error laps (B) and speed index (C) in Reversal sessions (*n* = 5 mice; symbols) in 20 laps before and after Reversal (colors, corridors as in A). (C) Filled symbols, correct lap; empty symbols, incorrect lap. (**D**) Performance before and after Reversal. (**E**) Top: Cumulative probability of difference in spatial information before and after Reversal in the striped (*n* = 810 cells, pooled) and checked (*n* = 724 cells) corridors. Symbols and whiskers, means ± SD. Bottom: Fraction of spatially tuned cells before and after Reversal. (**F**) Top: Cumulative probability of corridor selectivity difference before versus after Reversal (*n* = 1182 cells). Bottom: Fractions of corridor-selective cells before and after Reversal (*n* = 5 mice). The first eight laps after Reversal were excluded from the analysis in (E) and (F). (**G**) Cross-sorted, normalized activity rate maps of spatially tuned cells before or after Reversal in the indicated corridors (top). (**H**) Normalized activity difference of corridor-selective neurons before and after Reversal. (**I** and **L**) Average lap-to-lap correlation around Reversal in the striped (I) and checked (L) corridors (thick line and shadow, means ± SD). (**J** and **M**) Left: PV correlation of rate maps calculated from odd versus even laps in the striped (J) and checked (M) corridors before and after Reversal (*n* = 5 mice). Right: PV correlation between the spatial maps before and after Reversal. (**K** and **N**) Diagonals of the PV-correlation matrices before versus after Reversal in the striped (K) and checked (N) corridors. Means ± SD, dark purple in (K) and black in (N), with shading. Orange/red (K) and light blue/yellow (N) lines indicate correlations between even and odd laps before and after Reversal. (**O** and **P**) PV correlations of spatial positions between the two corridors before (O) and after (P) Reversal. Symbols and whiskers in (D) and [(E) and (F), bottom], means ± SE. Statistical analysis: table S1. Bef, before; Aft, after.

Next, we sought to investigate the dynamics of spatial and contextual representations under conditions when a context change occurs without any alterations in the visual attributes of the virtual environment. To this end, in five mice that already reached expert performance in the Pattern condition, we performed uncued intrasession reward reversal, i.e., we swapped which of the two corridors was rewarded, while the pattern and the color of the corridor pair remained the same (“reversal”, Figs. 1, B and C, 7A).

Upon the reversal of the reward contingency, there was no sudden change in the behavior of the animals and consequently the performance (ratio of correct laps) dropped immediately (Fig. 7, B and D; and fig. S8, C and D). Behavioral adaptation started only 3 to 10 laps after the Reversal and varied among the individual mice (Fig. 7, B and C); however, it generally took relatively long for the animals to reach expert level again (fig. S1B).

The number of active cells did not change significantly after the Reversal (before, 671 ± 53; and after, 615 ± 80; *P* = 0.224, Wilcoxon test). However, when comparing the activity of CA1PNs before and after the Reversal (excluding the first eight laps after the Reversal), we found that the fraction of spatially tuned and corridor selective cells decreased substantially both in the striped and the checked corridors (Fig. 7, E and F). The position- and corridor-dependent activity of the cells that were tuned before Reversal reorganized, but to a lesser degree than upon Switch (Fig. 7, G and H, cf Fig. 6, G and H; *P* < 0.001 for comparison to all respective changes in Switch, Mann-Whitney tests; see fig. S9B for example cells).

The population activity also reorganized after reward reversal. Lap-to-lap correlations decreased in both corridors (Fig. 7, I and L), spatial PV correlations decreased in the same corridors before versus after the Reversal, and the new representation had only a low correlation with the original activity (Fig. 7, J, K, M, and N). Similar to the Switch, the original decorrelation of the U and R corridor representations near and at the RZ (characteristic for the HIGH sessions; Fig. 4, F and N) disappeared, and the correlation of the activity in the two corridors became more uniform along the track (Fig. 7, O and P).

While the general characteristics of reorganization by the Switch and the Reversal manipulations were similar, the time course of the changes appeared to be different: In contrast to the abrupt change by the Switch, upon Reversal a more gradual decrease of lap-to-lap population activity correlation could be observed in both corridors (Fig. 7, I and L). Similarly, correlation of the population activity in individual laps to the average “template” activity showed a relatively slow decrease after Reversal in both corridors (fig. S8, E to H). On the other hand, the low correlations to an after-reversal template indicated no rapid emergence of a new activity map (fig. S8, G to H). Notably, we found that the original corridor representations were more preserved (higher correlations to the BEFORE template) in the error laps after the Reversal, compared to that in correct laps (fig. S8, E and F), suggesting that the hippocampal representation of the context is closely associated with the behavioral response. In conclusion, even in physically unchanged environments, reversal of the reward contingency led to substantial reorganization of the spatial and context-selective representations in CA1.

## DISCUSSION

Location- and context-specific activity patterns of hippocampal neurons are thought to play a fundamental role in spatial and contextual memory, yet how the two types of representations develop, coexist, and interact is not well understood. The spatial code is believed to appear quickly to enable the rapid formation of episodic memories embedded in their spatiotemporal context (*16*, *38*). However, context can be ambiguous and uncertain—a latent variable—and, often, the animal has to infer the task-relevant contextual features during interactions with the environment (*24*, *25*).

To study dynamic and relational changes of spatial and contextual representations in the hippocampus, we developed a head-fixed virtual reality paradigm that required both navigation along a virtual track and rule learning based on nonspatial features of the environment (*32*, *33*). There were several key features of our paradigm: (i) Only one type of virtual corridor was rewarded in one contingency; (ii) to avoid repetitive behavior, corridors were presented pseudorandomly, so no memory of the previous trial was needed but elevated attention was required to perform the task correctly; (iii) reward was delivered only after self-initiated licking at the RZ and the duration of the timeouts in virtual gray zones provided feedback about the mouse’s action in the previous lap; (iv) Head fixation and running on a linear track ensured no difference in trajectory, head direction, or nonvisual cues associated with each corridor.

We first studied the emergence of context-dependent hippocampal spatial representations during learning a contextual-spatial discrimination task. As the animals learned the task, the activity of the CA1PN population was initially dominated by coding the spatial structure of the corridors but did not discriminate well between them. Yet, the initial spatial code was not uniform but enriched at two behaviorally relevant areas (the start and the end of the corridors), suggesting that it adapted to support goal-oriented navigation to the RZ (*48*–*50*). As mice gradually learned to discriminate the valence of the corridors, the representations became more refined with the emergence of a large subset of CA1PNs with corridor-specific spatially tuned activity. As may be expected, these cells were most enriched near and at the RZ in R (*41*, *50*–*52*), allowing the most precise decoding of position and corridor ID from neuronal activity when the animal was at this location. While these results are consistent with previous studies showing adaptation in CA1 representations to overrepresent relevant spatial locations, they provide several intriguing insights.

First, a spatial representation that could relatively well code the animal’s position developed quickly although our virtual corridors contained very few location-specific visual cues (e.g., the end of the patterned zone), indicating that this process is robust against impoverishment of landmarks and of sensory modalities.

Second, changes in the proportions of cells with different tuning properties from LOW to HIGH indicate that the gradual enlargement of the corridor-specific cell population occurred in part by recruiting previously untuned cells and, in part, at the expense of the spatially tuned only cell population. The activity of individual cells that were tracked during behavioral adaptation from LOW to HIGH generally became suppressed in one of the corridors during learning and amplified in the one that they became selective for, similar to previous observations (*37*, *53*). These findings point toward a variety of plasticity processes involved in the development and refinement of the representation (*50*, *54*–*57*). Further experiments and analysis, investigating the characteristics of the lap-to-lap changes in activity of individual CA1PNs systematically during the complete course of learning, could shed light on these mechanisms (*58*).

Third, while the increased density of RZ-related cells in HIGH sessions is consistent with previous results and may reflect in part reward-related behavioral adaptation (*47*, *50*, *52*, *59*), we observed accumulation of corridor-selective cells around the reward location also in the U corridor. Notably, the emergence of these cells was not accompanied by zone-specific behavioral changes (as the animals already learned not to substantially decelerate and lick at this location), suggesting that the specific activity of these neurons represents abstract context-related variable(s) rather than being directly associated to reward related behavior.

Fourth, when the mice progressed from Color to Pattern discrimination (a second task with a novel rule to learn), the CA1 representation reset to its initial state with regard to the proportions of spatially tuned and corridor-selective PCs in both corridors. After the reset the gradual learning process was accompanied by a very similar development from an almost purely spatial representation to corridor-selective spatial code by the emergence of corridor-selective spatially tuned cells in the new corridor. This suggests that the progression from pure spatial to discriminative contextual coding is not due to certain specific experimental variables associated with learning in the Color condition (e.g., the type of nonspatial information (color) used for discrimination or the preceding shaping process) but is rather a common feature of hippocampal neuronal adaptation during the learning of complex tasks, where the timescales of the processes likely depend on the difficulty to infer the nonspatial contextual variables that are needed to be used for successful behavior.

It has been claimed that hippocampal representations are hierarchically organized (*45*, *46*), such that changing the context also alters the representation of location, item, or valence in a context-dependent navigational (*29*) or object association task (*46*). However, in these studies, different contexts were also associated with different boxes or reward locations, making it hard to separate the effect of context from other confounding factors. To examine the possibility of hierarchical versus independent representations of the pairs of corridors, we performed two manipulations during an imaging session in expert mice: context switch and reward reversal. Switching the task condition by changing the wall pattern of one of the corridors resulted in a notable, fast reorganization of the neuronal code, not only in the (novel) rewarded but even in the unchanged U corridor. The representational changes were highly similar in the two corridors, despite the large differences in their valence and in the alterations in the behavior in them. This result is consistent with hierarchical task representation at three distinct levels (rule, corridor, and position), where changing the rule (the top of the hierarchy) is expected to induce remapping at all lower levels. As a caveat, our mice have not learned the novel rules at the time of the rule change; therefore, we could not examine transitions between mature representations; further experiments with switches between previously acquired task rules would be ideal to conclusively assess the structure of the hippocampal spatial-contextual code and its relationship with behavioral adaptation (*46*, *60*). Nevertheless, the abrupt reorganization of the representation of the unchanged corridor clearly argues against the independent representations of the two corridors.

As an alternative possibility, we considered the question whether the animals remained engaged in the task after the change. It has been reported that voluntary disengagement from a virtual goal-oriented navigation task can lead to degradation of the hippocampal spatial code (*61*, *62*). Disengagement manifests as a sudden change in behavior when mice switch to stereotypical fast running without slowing down and licking near the previously learned RZ and is not directly induced by lower reward expectation (*62*). Disengagement could be occasionally observed in our well-trained mice as well (fig. S11), typically at the end of long behavioral sessions, similar to that reported (*61*). However, several considerations argue against disengagement to explain our results. The switch was carried out relatively early in the HIGH session, whereas disengagement occurred typically after >400 laps. The behavior of the four mice was variable in the first ~20 laps after the switch: Some mice kept licking, and, in most animals, the running speed was relatively variable across the first 20 laps, unlike in typical disengagement. Importantly, despite their variable behavioral responses, the changes in neuronal representations upon switch were almost immediate and qualitatively similar across mice. While, for these reasons, we believe that the observed effects were not due to disengagement, a change in the internal state or attention level of the animal by the switch is likely and could contribute to or facilitate creating less stable and more plastic hippocampal representations. These changes may promote building a novel contextual representation by incorporating relevant environmental features and sensory details.

In contrast to the switch experiment, reversal of the reward contingency in the two corridors led to a temporally more gradual change in representations. This slower adaptation may be intuitive because (contrary to the switch) there was no change in the available sensory cues in any of the corridors. Intriguingly, we found that the corridor representation was related to behavioral performance, as error laps after the reversal (when the animal persevered with the original, now wrong behavior) exhibited higher correlations with the prereversal representations than correct laps. These results are consistent with some previous observations (*62*), although other studies found more moderate representational changes after reward reversal (*33*, *60*, *63*) or task change (*46*, *60*, *64*).

Several features of our experimental conditions may affect the observed results and need to be emphasized. First, spatial navigation in virtual reality is less realistic and head fixation reduces motion information, which altogether could lead to less stable representations than during real world navigation (*65*). Second, throughout the whole experiment, including initial training, we presented the two corridors comprising the task in a randomized order, which may potentially facilitate formation of a conjunctive, hierarchical representation of the corridors. It is possible that training corridors in separate blocks [as often used during training in other studies; (*18*, *32*, *33*, *66*)] would rather induce an individual corridor to develop a specific behavioral pattern and an associated mature or stable independent neuronal code. The importance of randomized or blockwise training will need to be addressed by future experiments [see also (*67*, *68*)].

In conclusion, our results indicate that, in this paradigm, in line with behavioral adaptation, an already present spatial code is complemented by a contextual code with experience. The sudden activity reorganization upon Switch of both spatially tuned and corridor selective activity suggests a hierarchical conjunctive encoding of task rule, corridor, and position by CA1PNs.

## MATERIALS AND METHODS

### Animals

Experiments were conducted in accordance with the guidelines of the European Communities Council Directive (86/609 EEC), the Hungarian National Scientific Ethical Committee on Animal Experimentation regulation no. AA2.0/2015, and the Animal Care and Use Committee of the Institute of Experimental Medicine and with the approval of the Committee for Scientific Ethics of Animal Research of the Government Office of Pest County Department of Food Chain Safety, Veterinary Office, Plant Protection and Soil Conservation Budapest under the project number PE/EA/676-7/2021. All efforts were made to minimize pain and suffering and to reduce the number of animals used. Adult (48 to 73 days old at the beginning of the experiments) heterozygous C57BL/6 J-Tg(Thy1-GCaMP6s)GP4.3Dkim/J mice (*69*) (Jackson Laboratory, stock no. 024275) crossed with wild-type C57/Bl6J mice (Jackson Laboratory, stock no. 000664) were used (*n* = 9, 4 females and 5 males). Mice were housed in a vivarium in groups (two to five mice per cage) on a reversed 12-hour light/dark cycle. Experiments were performed during the dark phase of the cycle.

### Surgery

At the age of 44 to 63 days, mice were surgically implanted with a hippocampal cranial imaging window and headpost, according to previously established protocols (*70*). Briefly, under Isoflurane (3.5 to 2%) anesthesia and with the application of local anesthetic (ropivacaine, 0.05 ml) subcutaneously, a 3-mm-diameter craniotomy was made in the exposed skull over the left dorsal hippocampus. After gentle removal of the dura, the underlying cortex was slowly aspirated with continuous irrigation with chilled Ringer solution until horizontal fibers of the corpus callosum were exposed. Special care was taken not to remove deep fibers running in the anteroposterior direction. A custom-made imaging cannula [3-mm diameter by 1.5-mm height; (*71*)] made by adhering (Norland optical adhesive #81) a 3-mm round glass cover slip (Deckglaser) window to a stainless-steel cylinder was inserted into the craniotomy. The imaging cannula was cemented to the skull with black dental acrylic (Unifast Trad with carbon powder added to the cement) along with a light but durable custom-designed titanium alloy (TiAl6V4) headpost for head fixation.

### Behavioral training

A minimum of 4 days after the implant surgery, mice were introduced to a water restriction protocol (keeping their weight above 80% of their predeprivation body weight) and hand habituation (Fig. 1C). After familiarization with the microscope and the lick port in a translucent box while being head fixed, mice were first trained to run on a cueless belt on a treadmill for water, by rewarding them with water drops of variable preset size and adjusting the distance between water delivery sites (“lick & run training,” for 3 to 4 days). Mice were then exposed to the virtual reality monitors, and reward zone training (“RZ training”) in the virtual environment started with gradually decreasing the number (from 9 covering the full length of the belt to 1) of cued RZs, visually indicated by blue drops over black background. In the next stage, a 75-cm-long gray wall with rhomboid pattern was introduced, and 1 drop of water reward was given in the black section after the patterned part of the corridor. Reward was initially indicated visually with the blue drop, which was removed once mice learned the RZ location. Water was initially delivered nonoperantly (mice automatically received water when they entered the RZ), but, in the final stage of the RZ training, mice were required to operantly lick to trigger water reward in the RZ. Lick & run and RZ training was performed in 15- to 20-min blocks and took altogether ~2 weeks. During this period, mice were also habituated to the scanner and shutter sounds during occasional checks of the imaging window.

### Virtual contextual go/no-go task

After RZ training, mice were introduced to the goal-directed contextual go/no-go task, in which they had to learn to discriminate between two visually distinct virtual corridors per condition, based either on their color or their pattern, and had to lick selectively in the uncued RZ of the R corridor (Fig. 1, B and C). Virtual linear tracks consisted of an 88.5-cm-long section with corridor-specific patterned walls followed by a 17.5-cm-long section with uniform black walls. The length of the RZ was 1/10th of the corridor (~10 cm) and was located in the black corridor section, before the end of the 106 cm-long virtual environment. Mice had to lick operantly in the RZ of the R corridor to trigger the delivery of one drop of water reward. When the mice reached the end of a virtual corridor, a gray timeout corridor (106 cm) was presented. Mice had to move to start the next lap: Following correct laps, they could leave the gray corridor after 0.5 s; following error laps, they could either run through the entire gray corridor (taking more than 2 s) or leave after 8 s. The two types of (U and R) corridors (separated by the gray corridor) were presented in a pseudorandom order to obtain similar numbers of laps for analysis from the two corridors. The LOW-MED-HIGH 2P imaging sessions lasted for 10 min with 31 to 261 laps run, while the Switch and Reversal sessions took up to 20 min. Both LOW-MED-HIGH and Switch/Reversal imaging epochs were embedded in longer behavioral sessions without imaging (30- to 50-min blocks, ~300 to 500 laps per behavioral session). Mice typically performed one or two sessions a day.

The experiment series (Fig. 1C) started with the Color discrimination task using purple striped as U and green striped as R corridor walls. When mice reached the expert learning criterion (performance at least 90% in 40 laps; taking 1 to 13 days of learning), they were exposed to the second task, Pattern discrimination, using purple striped as unrewarded, and purple checked as R corridor walls. Mice were required to again reach 90% performance over 40 laps (which took 1 to 8 days) in this second condition to proceed to the third, Reversed task. In this latter condition, the purple striped corridor was rewarded, while the purple checked was the U corridor wall. In some cases, more than one learnt session of a condition was recorded for stability control. In a subset of animals, we switched between conditions (from Color to Pattern or from Pattern to Reversed) within one imaging session. These experiments are presented in this study as Switch and Reversal sessions, respectively.

### Data inclusion criteria

Together, 289 imaging sessions were recorded from 11 mice performing the virtual contextual go/no-go task presented in this manuscript. One mouse was excluded from the analysis because of the detachment of the hippocampus from the imaging window over the course of the experiments. We also excluded another mouse that had bad imaging quality (weak signals because of a water leak from below the objective) in its recorded HIGH-performance session of the Color discrimination condition. In the imaging analysis, we used 76 imaging sessions from nine mice, which met the following inclusion criteria. (i) The animals had at least one LOW- and one HIGH-performance (see the “Behavioral analysis” section) epoch from at least one condition (Color, Pattern, or Reversed). MED sessions could be missing in some set of recordings if transition to HIGH happened in one session after a LOW session. (ii) An epoch had to contain at least 30 imaged laps to be analyzed. (iii) Stable and good quality optical access to CA1PNs was needed throughout the imaging epoch. Two male mice underwent pretraining of Color discrimination using shorter textured wall segments, but without showing any performance improvement; therefore, they continued with the original task described above. Not all mice were analyzed for imaging in all conditions: We used rn18, rn19, rn20, rn22, rn38, rn39, rn42, and rn45 in Color discrimination; rn18, rn20, rn22, rn38, rn39, rn42, rn44, and rn45 in Pattern discrimination; rn38, rn39, rn42, rn44, and rn45 in Reversed Pattern discrimination; rn38, rn42, rn44, and rn45 in Switch; rn38, rn39, rn42, rn44, and rn45 in Reversal sessions; rn38, rn42, and rn45 in the Reswitch experiment; and rn22, rn38, rn39, rn44, and rn45 for tracking the same cells across multiple epochs. In the case of Switch/Reversal sessions, we also required that sessions have more than 30 laps before the Switch/Reversal for analyses. Within each behavioral session, we analyzed laps within imaging epochs selected to reach LOW-MED-HIGH performance criteria (see the “Behavioral analysis” section) based on licks in the RZ. In four of the nine mice, after 375 to 619 laps (median of 454 laps in *n* = 9 sessions), we observed signs of disengagement from the task based on licking behavior and velocity change (fast running without anticipatory slowing, no licking) (*61*). The number of laps run before Switch/Reversal was lower, ranging from 49 to 527 laps (median of 214 laps in *n* = 18 sessions) and fell in the above disengagement range in only two cases.

### Behavioral apparatus and virtual reality

The behavioral setup (Luigs & Neumann, Fig. 1A) consisted of a treadmill covered with a cueless black fabric belt (94 cm long), surrounded by three monitors at 90° angles with each other projecting linear virtual environments. A lick port (a waterspout combined with a lick sensor) was used for lick detection and water dispersion. Locomotion (speed) was recorded by tracking the rotation of the treadmill wheel using a magnetic rotary encoder. For synchronization of behavioral recordings and image acquisition, transistor-transistor logic (TTL) pulses of increasing lengths were generated on the behavioral computer and were recorded by PrairieView (Bruker) on the imaging computer for offline pairing. Linear virtual environments were constructed using a modified version of the script written by Luigs & Neumann in Ogre. Images for the textured corridor walls were generated in Adobe Photoshop. Behavioral apparatus was controlled using LabVIEW graphical programming environment.

### In vivo 2P imaging

Calcium imaging in head-fixed, behaving mice was performed using a 2P microscope (Bruker Investigator) equipped with an 8-kHz resonant scanner and a Ti:Sapphire laser (Chameleon Vision II, Coherent) tuned to 920 nm. For image acquisition, we used a Nikon 16× LWD water-immersion objective (0.8 numerical aperture, 3-mm working distance). Stray light from the virtual reality monitor was blocked from reaching the photomultiplier tube (PMT) by a custom-designed, 3D-printed black plastic cone that was fitted over the outer rim of the head plate and connected to the lens tube with a thick black light-blocking rubber tube. To adjust the angle of the imaging window to the plane of the front lens of the objective, we set the angle of the head-fixed mouse’s head with two goniometers (Edmond Optics). Fluorescent signals were collected by a GaAsP PMT (Hamamatsu) at 1× digital zoom, acquired as 512 pixels by 512 pixels single-plane images with resonant scanning at ~30 Hz.

### Behavioral analysis

The analysis of mouse behavior during the virtual contextual go/no-go task was performed using custom Python scripts. To define discrimination between the U and the R corridors and to quantify behavioral adaptation to the task in the two corridor types separately, first, we divided each corridor into 50 spatial bins and identified a 5-bin-long prezone (PZ) immediately before the RZ and a 5-bin-long control zone (CZ), starting 25 bins before the PZ. We calculated two types of metrics based on lick rate [L(t)] and average running speed [v(t)] (Fig. 1E): (i) Lick and speed indices (*61*) were calculated within both corridors separately as $\left(x_{\text{PZ}}\;-\;x_{\text{CZ}}\right)/\left(x_{\text{PZ}}\;+\;x_{\text{CZ}}\right)$; and (ii) lick and speed selectivity between corridors: $\left(x_{\text{PZ}}^{\text{rew}}\;-\;x_{\text{PZ}}^{\text{unrew}}\right)/\left(x_{\text{PZ}}^{\text{rew}}\;+\;x_{\text{PZ}}^{\text{unrew}}\right)$, with $x_{\text{PZ}}^{\text{rew}}$ and $x_{\text{PZ}}^{\text{unrew}}$ being either the lick rate or the average speed in the PZ of the R and U corridors, respectively.

To evaluate learning performance (perf), we calculated correct and incorrect lick choices (see the “Virtual contextual go/no-go task” section) in each lap of a session, as well as in the U and R corridors, separately. On the basis of the overall behavioral performance (percentage of correct laps) during one imaging session, we selected a LOW (perf < 0.65)– and HIGH (perf > 0.84)–performance epoch with at least 30 laps for analysis in each animal in both the Color and the Pattern discrimination task. For analysis of intermediate (MED) sessions (fig. S3), we selected an epoch with performance approximately in the middle between the LOW and HIGH epoch of the given animal. Note that some mice did not have appropriate MED sessions. For the analysis of the number of trials needed to learn the task in the different conditions (Fig. 1D and fig. S1B), we also included an animal that was excluded from the imaging analysis (as described previously in the “Data inclusion criteria” section).

### Analysis of neural Ca^2+^ activity

#### Ca^2+^ data preprocessing

The acquired 2P recordings were preprocessed for further analysis using open-source software packages and custom-written scripts in Python (fig. S2A). Motion correction of the imaging data, segmentation of regions of interest (ROIs), and extraction of raw GCaMP6s fluorescent Ca^2+^ signals were performed from registered movies using Suite2p (version 0.7.1.) (*72*). ROIs of putative CA1PNs were manually curated to include ROIs falsely identified as “not cell” by the algorithm and to exclude (i) ROIs with low signal-to-noise ratio, (ii) multiple cells coregistered as one ROI (based on shape and size), and (iii) ROIs with contaminated activity (based on overlap between neighboring ROI signals) in a highly conservative manner, using Suite2p graphical interface.

Active cells were defined as cells having at least one Ca^2+^ event per minute (with amplitude larger than the detection threshold, defined as three times the cell-specific noise SD) separated by at least 5 s. To measure the amplitude, we first calculated the relative fluorescence change [$\Delta F/F\;=\;\left(F\;-\;F_{0}\right)/F_{0}$], where $F_{0}$ is the mode of the raw fluorescent signal *F*, and then smoothed the ΔF/F signal with a 100-ms Gaussian kernel. To measure the cell-specific noise SD, we selected baseline (“resting”) *dF*/*F* trace segments lasting for at least 1 s, which had a low total count of inferred spikes (<20 spikes/s). Raw fluorescence traces were deconvolved using OASIS (*73*) with τ=0.8 s to estimate the underlying spiking activity. The inferred spikes were used for all other analyses.

#### Extracting spatial and corridor-specific activity

We restricted our analysis to periods when the animal was running (velocity of >5 cm/s) in one of the corridors (excluding gray timeout zones) and focused on active cells (see the “Ca^2+^ data preprocessing” section). Note that neuronal Ca^2+^ signals were not analyzed when mice stopped in the RZ of the R corridors. The spikes inferred from Ca^2+^ activity were spatially binned (50 bins of 2.12-cm width), averaged across the three neighboring spatial bins, and summed across laps, and, then, the total spike count was divided by the time spent in each bin to estimate the position-dependent activity rate (“tuning curve”) of individual cells. Tuning curves were calculated separately in the two corridors. When comparing the spatial tuning of the same cell in different conditions (e.g., Fig. 4A), we estimated tuning curves independently for even and odd laps.

We computed several metrics to identify all spatially tuned or corridor-selective cells in our recordings. Specifically, spatial tuning was evaluated by calculating three different parameters: (i) spatial reliability, i.e., the average Pearson correlation between the spiking in individual laps and the neuron’s tuning curve; (ii) tuning specificity, i.e., the length of the corridor divided by the width (SD) of the tuning curve; and (iii) spatial information content, i.e., the estimated mutual information between position and the tuning curve (*74*). To characterize corridor-selective activity, we calculated corridor selectivity, i.e., $\left(r^{\text{rew}}\;-\;r^{\text{unrew}}\right)/\left(r^{\text{rew}}\;+\;r^{\text{unrew}}\right)$, where $r^{\text{rew}}$ and $r^{\text{unrew}}$ are the total number of spikes in the R or U (respectively) corridor divided by the total time spent there. We calculated selectivity both for the whole corridor and in four different zones of the corridor, i.e., (pattern) P1, bins 0 to 13; P2, bins 14 to 27; P3, bins 28 to 41; RZ, bins 42 to 45 or 45 to 48, separately.

In Figs. 6 (E and F) 7 (E and F), the change in the quality of the spatial representation was assessed by measuring the spatial information and corridor selectivity of cells sufficiently active (average estimated firing rate > 1 Hz) both before and after the task change. Significance of the spatial tuning and corridor selectivity of the cells was identified by a shuffle analysis. Specifically, we created 1000 shuffled rate maps by circularly shifting the spiking activity of each cell relative to the true position of the mouse and then dividing it into five chunks of at least 500 frames whose order was randomly permuted. Cells with significantly higher values in at least one of the spatial metrics or selectivity (*P* < 0.05) were considered as spatially tuned in the given corridor or as corridor selective. We used the Holm-Bonferroni method to correct for multiple statistical tests.

#### Cross-correlation of population rate maps

The similarity of the spatial maps in two different conditions (rewarded versus unrewarded corridor, even versus odd laps, etc.; Fig. 4B, C, F, G, J, K, N, O; Fig. 5D to E; Fig. 6I to P; and Fig. 7I to P) was characterized by the correlation matrix between the population vectors with elements $M_{\mathrm{ij}}\;=\;\text{corr}\left({\mathrm{PV}}_{i}^{A},{\mathrm{PV}}_{j}^{B}\right)$ where ${\mathrm{PV}}_{i}^{A}$ is a vector containing the firing rate of all active cells in spatial bin i measured in condition A. We quantified the similarity of the spatial code in the two conditions by the diagonal of this matrix.

#### Lap-to-lap correlation of population activity

To characterize the changes in the population activity upon context switch or reward reversal, we calculated the lap-to-lap correlation of the population activity. Specifically, we estimated the spatial activity of all active cells in each lap and calculated the correlation between the activity vectors between a pair of laps as $C_{\mathrm{ij}\;}=\;\text{corr}\left({\mathrm{AV}}_{i},{\mathrm{AV}}_{j}\right)$ where ${\mathrm{AV}}_{i}$ is a vector containing the firing rate of all active cells in all spatial bins in lap i. We calculated the correlation matrix C using 20 laps before and after the Switch (Reversal) event in both corridors. Figures 6 (I and L) and 7 (I and L) show the average correlation of lap *j* with other laps, $C_{i}\;=\;\frac{1}{40}\sum_{j}C_{\mathrm{ij}}$, centered on the Switch (Reversal) event.

#### Lapwise correlation to template population activity

In fig. S8 (E to H), we characterized the similarity of the population activity in a given lap to several activity templates by the correlation $B_{i}\;=\;\text{corr}\left({\mathrm{AV}}_{i},{\mathrm{TV}}^{B}\right)$ where ${\mathrm{AV}}_{i}$ is the activity vector in lap i as defined above, and ${\mathrm{TV}}^{B}$ is the template vector calculated by concatenating the tuning curves of all active cells. We estimated the tuning curves for the template vector using all laps before or after the Switch (Reversal) excluding 20 laps immediately around (before and after) the Switch.

#### Tuning curve correlations

To assess the stability of the spatial tuning across multiple switches (fig. S10, repeated switch), we calculated the correlation between the tuning curves of individual cells estimated from odd or even laps in different imaging sessions. As a baseline, we also calculated the correlation between the tuning curves estimated from odd versus even laps of the same session.

### Bayesian decoding of spatial position/corridor identity

For the decoding analysis, we used the spikes inferred from the activity of the manually selected ROIs. We first removed all frames that belonged to the gray timeout zones between the corridors and laps with less than 40 frames (usually incomplete laps at the beginning or the end of the imaging session). We also removed frames where the speed of the animal was below 3 cm/s. The spikes of the cells were then smoothed with an 11-frame-long uniform kernel and binarized with a threshold of 0.5. The position of the animal was divided into 50 spatial bins within each corridor, omitting the first 2% of the corridors (where the animal could not have information about the corridor identity). To compare different animals and imaging sessions with various numbers of simultaneously recorded neurons, we used 400 neurons in each decoder and averaged the results across 20 independent subsamples. The firing probability of neuron i in the spatial bin $x_{j}$ in corridor $c_{k}$, $P\left(s_{i}|x_{j},c_{k}\right)$, was estimated as the fraction of frames with spikes in a subset of the frames (training data), i.e.,

$$P\left(s_{i}|x_{j},c_{k}\right)\;=\;\sum_{x=x_{j},c=c_{k}}s_{i}\left(x,c\right)/T_{\mathrm{jk}}$$

where $T_{\mathrm{jk}}$ is the number of frames in the spatial bin $x_{j}$ in corridor $c_{k}$. The decoder was evaluated on the remaining part of the data (test data) by calculating the posterior probability in each location bin in both corridors: $P\left(x,c|s\right)\propto \prod_{i}P\left(s_{i}|x,c\right)P\left(x,c\right)$, where we used a uniform prior over position and corridors. For position or corridor identity decoding, we marginalized the posterior across corridors or positions, respectively

$$P(x|s)\;=\;\sum_{c}P\left(x,c|s\right)$$

$$P(c|s)\;=\;\sum_{x}P\left(x,c|s\right)$$

We used the median of the marginalized posterior as the predicted position. For decoding results shown in Fig. 4 and fig. S6, we performed 10-fold cross-validation using 90% of the frames as training data and the remaining frames for testing.

### Cross-registration of ROIs

To calculate tuning-curve correlations between the Ca^2+^ activities of individual cells recorded during two separate imaging epochs, first, we concatenated the image series acquired in the two epochs and ran Suite2p on it. Then, we compared the traces we got from this concatenated recording to the ones from the individual epochs to identify the same cells. A pair of ROIs in the two different epochs was considered to be related to the same cell only if both their signals had a higher than 0.958 correlation to the corresponding part of the signal from the concatenated recording.

For the analysis in Fig. 5, we used imaging epochs that were acquired without changing head fixation (i.e., without taking out the mice from the setup between recordings). To examine the activity of the same individual cells tracked across LOW- versus HIGH-performance epochs, we used 2P recordings of the same lap numbers, taken from the same FOV. To analyze neuronal activities, we included all ROIs that were sufficiently active in at least one of the epochs and used this common pool of coregistered cells in the corresponding LOW and HIGH epochs.

For figs. S9 and S10, we only included ROIs that were previously accepted as cells during the manual curation test for both epochs. In the rare case, when an ROI had multiple pairs from the other epoch, these pairs were not included in further analysis.

### Statistics

General linear model with repeated measures analysis of variance (ANOVA) was applied using within-subject factors including performance, corridor, and position (zone), depending on the experiment. Tukey’s test was applied for post hoc comparisons. The different conditions (Color, Pattern, and Reversed) were analyzed separately. Nonparametric Mann-Whitney or Wilcoxon test was applied for unpaired or paired comparisons, respectively. Statistical analyses were performed using the Statistica software (version 14.0.1.25, Tibco), except chi-square test performed in Python. The statistical analyses of results presented in the figures are summarized either in the corresponding figure captions or in table S1.

### Software

For the generation of figures, Adobe Illustrator, Adobe Photoshop, and Origin were used along with Python. Figure 5F was prepared using SankeyMATIC (sankeymatic.com).
