# Supplementary material for: Adaptation of hippocampal spatial and contextual representations to task structure
Source: Sci Adv. 2025 Nov 14;11(46):eadu4899. doi: 10.1126/sciadv.adu4899 (PMC12617488; doi:10.1126/sciadv.adu4899)
Supplement: Supplementary file 1 — Figs. S1 to S11 Legend for table S1 [file sciadv.adu4899_sm.pdf]

Science Advances
AAAS

Supplementary Materials for

Adaptation of hippocampal spatial and contextual representations to task structure

Rita Nyilas *et al.*

Corresponding author: Balázs B. Ujfalussy, makara.judit@koki.hun-ren.hu; Judit K. Makara, ujfalussy.balazs@koki.hun-ren.hu

Sci. Adv. **11**, eadu4899 (2025)
DOI: 10.1126/sciadv.adu4899

The PDF file includes:

Figs. S1 to S11
Legend for table S1

Other Supplementary Material for this manuscript includes the following:

Table S1

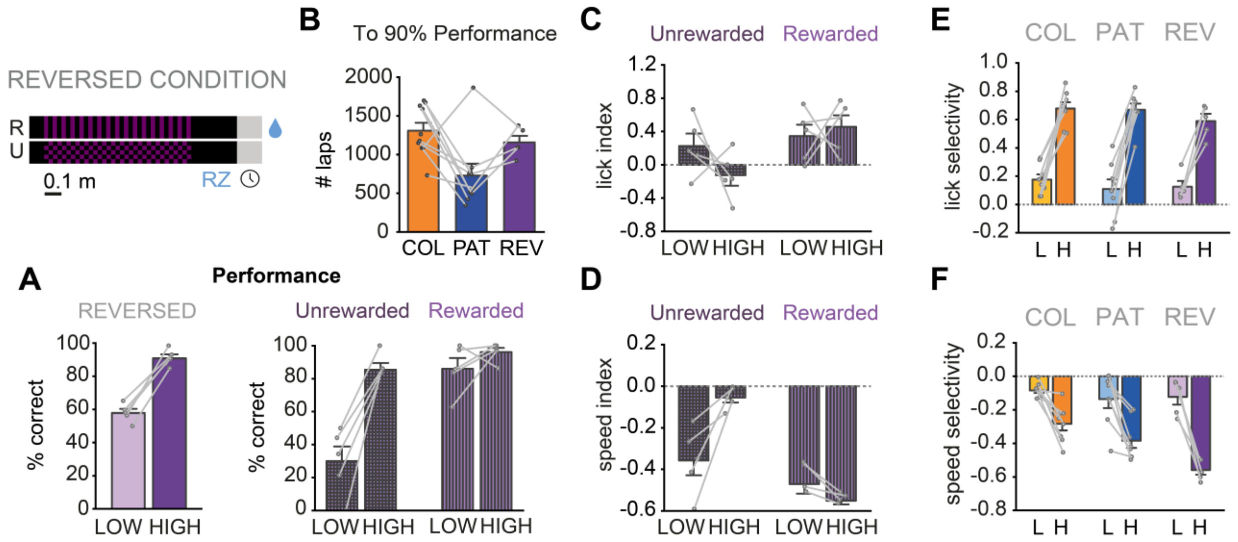

Fig. S1. Behavioral adaptation to the Reversed task.

(**A**), Performance in LOW vs. HIGH sessions. Left, % of correct laps (Wilcoxon test: p=0.043). Right, % of correct laps in U and R corridors separately in LOW and HIGH sessions. 2-way repeated measures (RM) ANOVA: performance (perf): p<0.001, corridor (corr): p=0.017, perf x corr: p=0.032). Connected dots correspond to individual mice (n = 5 mice), bars show mean ± SE across mice. Note that after Reversal, behavioral adaptation initially varied among individual mice (Fig. 7B-C), but eventually all mice started to lick in both corridors, as in typical LOW sessions.

(**B**) Number of laps to reach 90% performance in the Color, Pattern and Reversed conditions. Connected dots correspond to individual mice, bars show mean ± SE across mice.

(**C-D**) Intra-corridor lick and speed index calculated separately in U and R corridors, as in Fig. 1 (lick index: 2-way RM ANOVA: perf: p=0.672, corr: p<0.001, perf x corr: p=0.021; speed index: perf: p=0.088, corr: p<0.001, perf x corr: p=0.002).

(**E-F**) Inter-corridor lick and speed selectivity between U vs. R corridors (see Fig. 1 and *Methods*), in the three sequential task conditions (Color, COL; Pattern, PAT; Reversed, REV) before (L) and after (H) learning. Reversed lick selectivity: p=0.043, speed selectivity: p=0.043, Wilcoxon test.

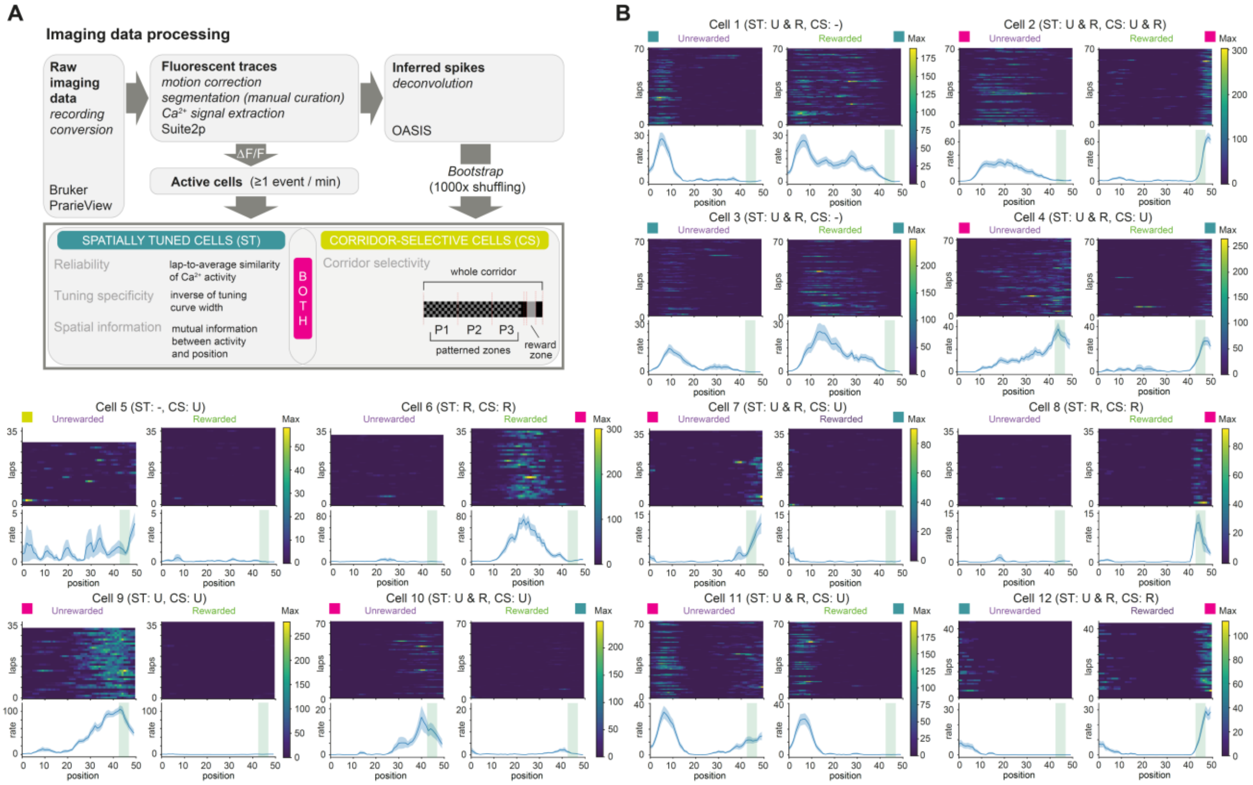

Fig. S2. Analysis workflow and example cell activities.

(**A**) Analysis workflow. Raw imaging traces were first motion corrected and ROIs were identified. Putative spikes were inferred from the fluorescent data and spatial tuning and corridor selectivity were determined for all active cells (see *Methods*).

(**B**) Example cells for spatially tuned and corridor selective activity. The lap-by-lap activity of each neuron is shown in both corridors above the mean ± SE of their estimated firing rate. Panel titles: ST: spatially tuned. CS: corridor selective. U: Unrewarded corridor. R: Rewarded corridor. e.g.: cell 4: ST: U & R, CS: U: spatially tuned in both corridors. Corridor selective with a higher firing rate in the Unrewarded corridor. Note, that a neuron can be selective for different corridors in different locations (e.g., cell 2).

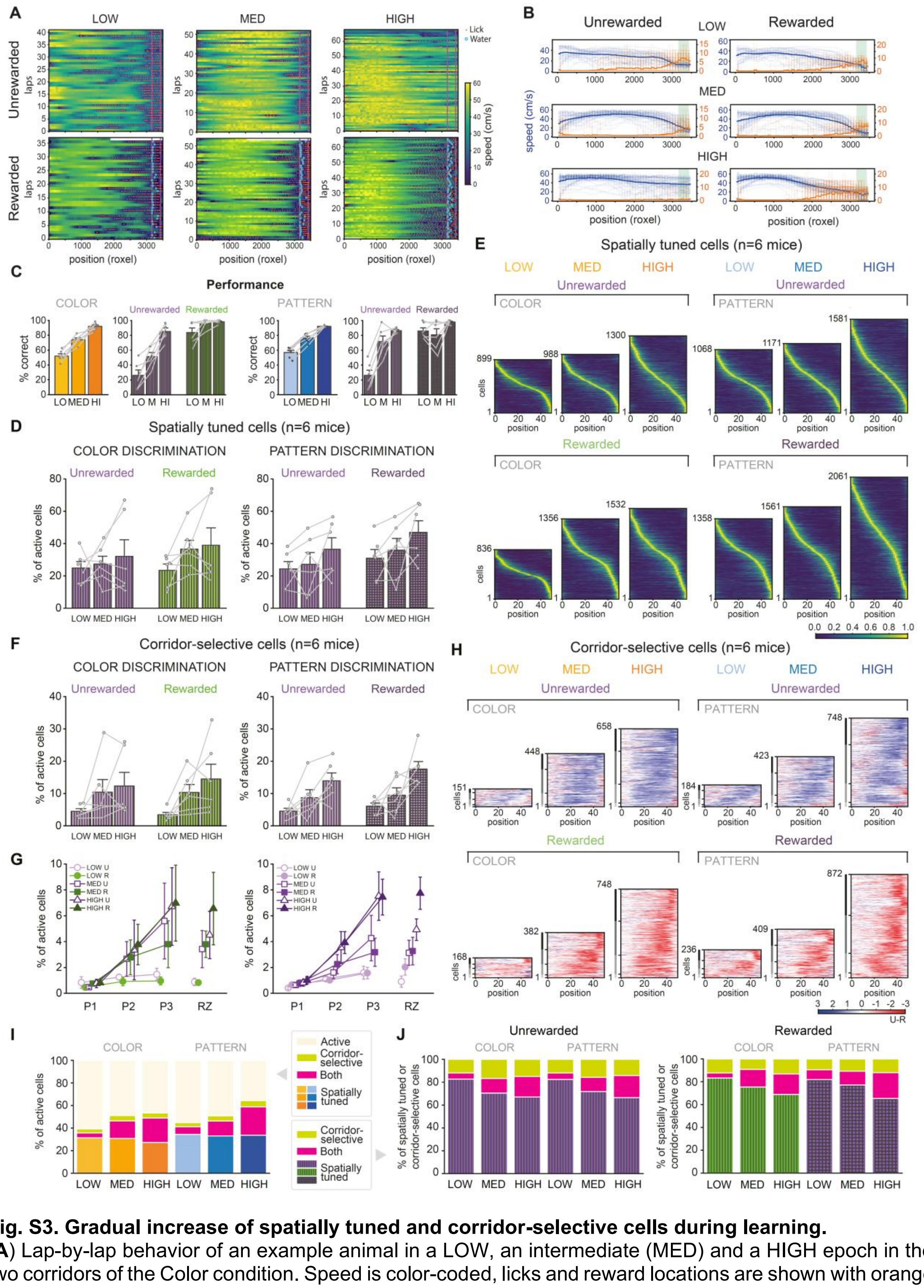

Fig. S3. Gradual increase of spatially tuned and corridor-selective cells during learning.
(**A**) Lap-by-lap behavior of an example animal in a LOW, an intermediate (MED) and a HIGH epoch in the two corridors of the Color condition. Speed is color-coded, licks and reward locations are shown with orange dots and blue circles, respectively.

(**B**) Individual laps (thin lines) and average (thick lines) speed (blue) and lick rate (orange) of an example animal in a LOW, a MED and a HIGH (HI) epoch in the two corridors.

(**C**) Summary of the performance of the animals in LOW (LO), intermediate (MED, M) and HIGH (HI) sessions in the Color and in the Pattern condition.

(**D**) Percent of spatially tuned cells in the Color and in the Pattern condition.

(**E**) Rate map of spatially tuned cells in the Color and in the Pattern condition.

(**F**) Percent of corridor-selective cells in the Color and in the Pattern condition.

(**G**) Percent of zone selective cells (significantly selective in the indicated zone, see Fig. 3D and *Methods* for zone definitions) of the U or R corridor in the Color and Pattern condition.

(**H**) Normalized activity difference of the corridor-selective cells in the Color and Pattern condition.

(**I**) Percent of cells with significant spatial tuning, corridor-selectivity, or both within all active cells.

(**J**) Percent of cells with significant spatial tuning, corridor-selectivity or both within all tuned or selective cells in LOW, MED and HIGH sessions (Color and Pattern condition). Data are from n = 6 mice in each task, whereas data presented in Fig. 1 (LOW and HIGH epochs) included data from 2 additional animals.

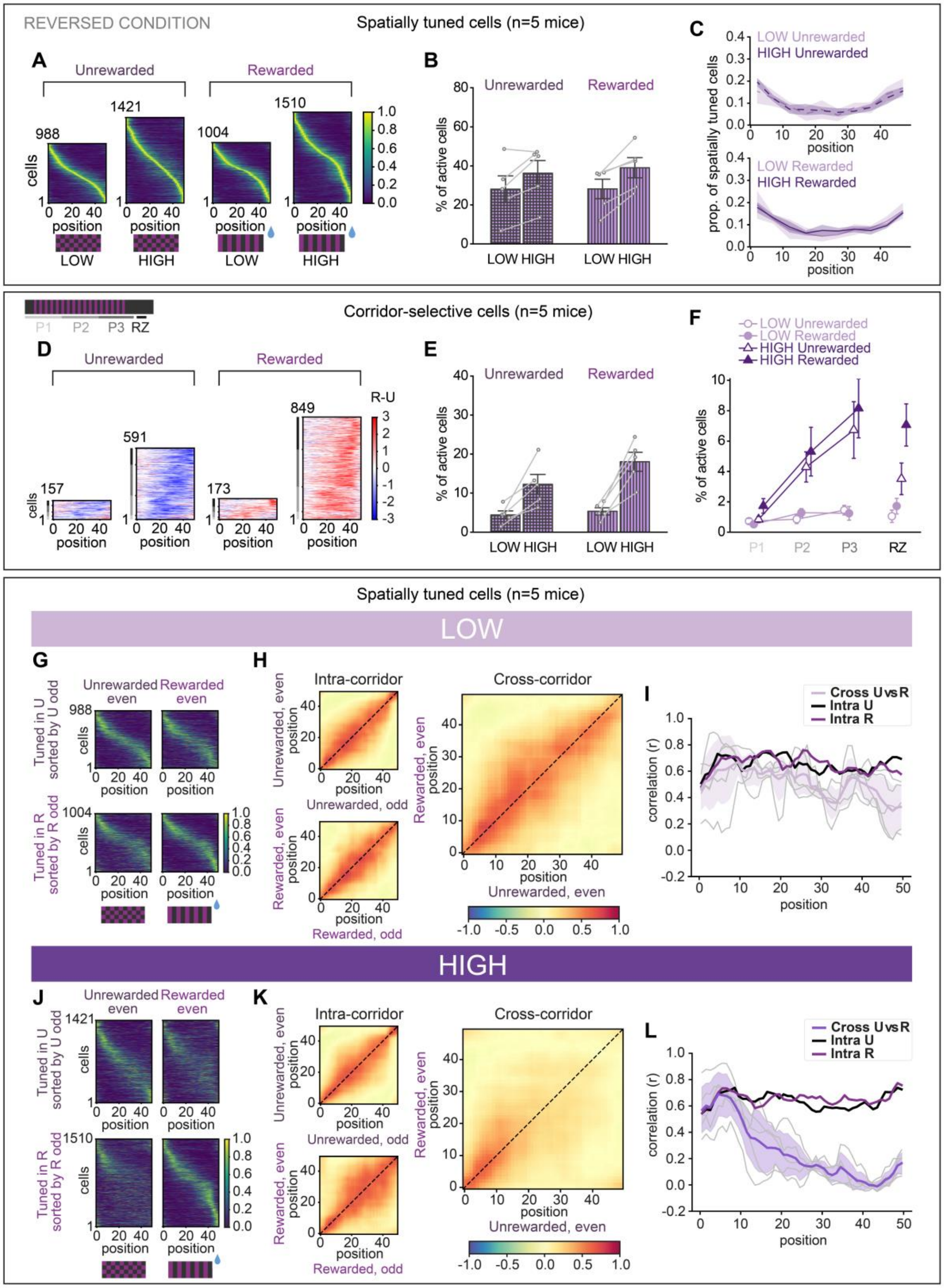

Fig. S4. Development of the neuronal representation during learning the Reversed task
(**A**) Color-coded normalized population activity ratemaps of CA1PNs with significant spatially tuned activity in Reversed condition in LOW and HIGH sessions. Cells were pooled from 5 mice and were sorted by the location of their peak activity.

(**B**) Percent of spatially tuned cells within all active cells. Data points, individual mice; bar plots, mean +/- SE (n=5 mice).
(**C**) Proportion of spatially tuned cells along 50 position bins of U and R corridors in LOW and HIGH sessions (mean +/- SD, n=5 mice).
(**D**) Normalised activity difference of the neurons selective for the Unrewarded (left) and the Rewarded (right) corridor. In each plot neurons are ordered by the location zone of their selectivity (shades of grey on the left, see inset on the top). Neurons selective in multiple zones are shown in each. Cells were pooled from 5 mice.
(**E**) as in (**B**), for corridor-selective cells.
(**F**) Percent of zone-selective cells (significantly selective in the indicated zone) of the U or R corridor in LOW and HIGH sessions (mean +/- SD, n=5 mice).
(**G, J**) Cross-sorted, color-coded normalised activity rate maps of cells with significant spatially tuned activity in the indicated corridors in LOW (**G**) and HIGH (**J**) sessions.
(**H, K**) Left, PV-correlation of spatial ratemaps calculated from odd vs. even laps in the U (upper) or R (lower) corridor. Right, PV-correlation between the spatial ratemaps in the two corridors in LOW (**H**), and HIGH (**K**) sessions (average across n=5 mice).
(**I, L**) The diagonals of the PV-correlation matrices between the U vs. R corridors of individual animals (grey, n=5) and their mean +/- SD (**I**: lavender, L: purple). Black and indigo lines represent the mean diagonal of the corresponding intra-corridor ratemap correlation matrices.
Statistical analysis: Table S1.

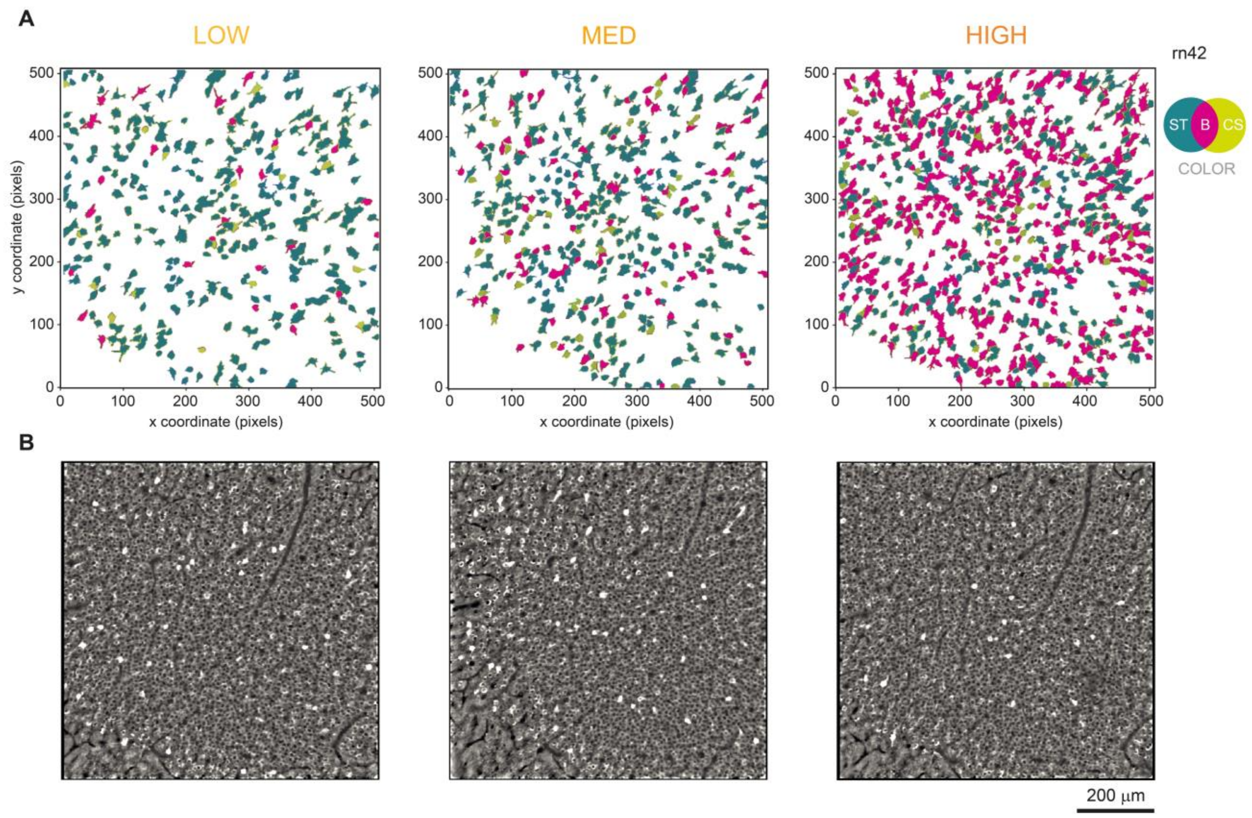

Fig. S5. Scattered distribution of tuned CA1PNs.
(**A**) Distribution of the cells with significant spatial tuning (ST, teal), corridor selectivity (CS, light green) or both (B, magenta) in a LOW, an intermediate (MED) and a HIGH (as indicated above) epoch in the Color condition of an example animal.
(**B**) Imaging field of view (FOV, mean enhanced Suite2p image at 920 nm) in the same epochs as in **A**.

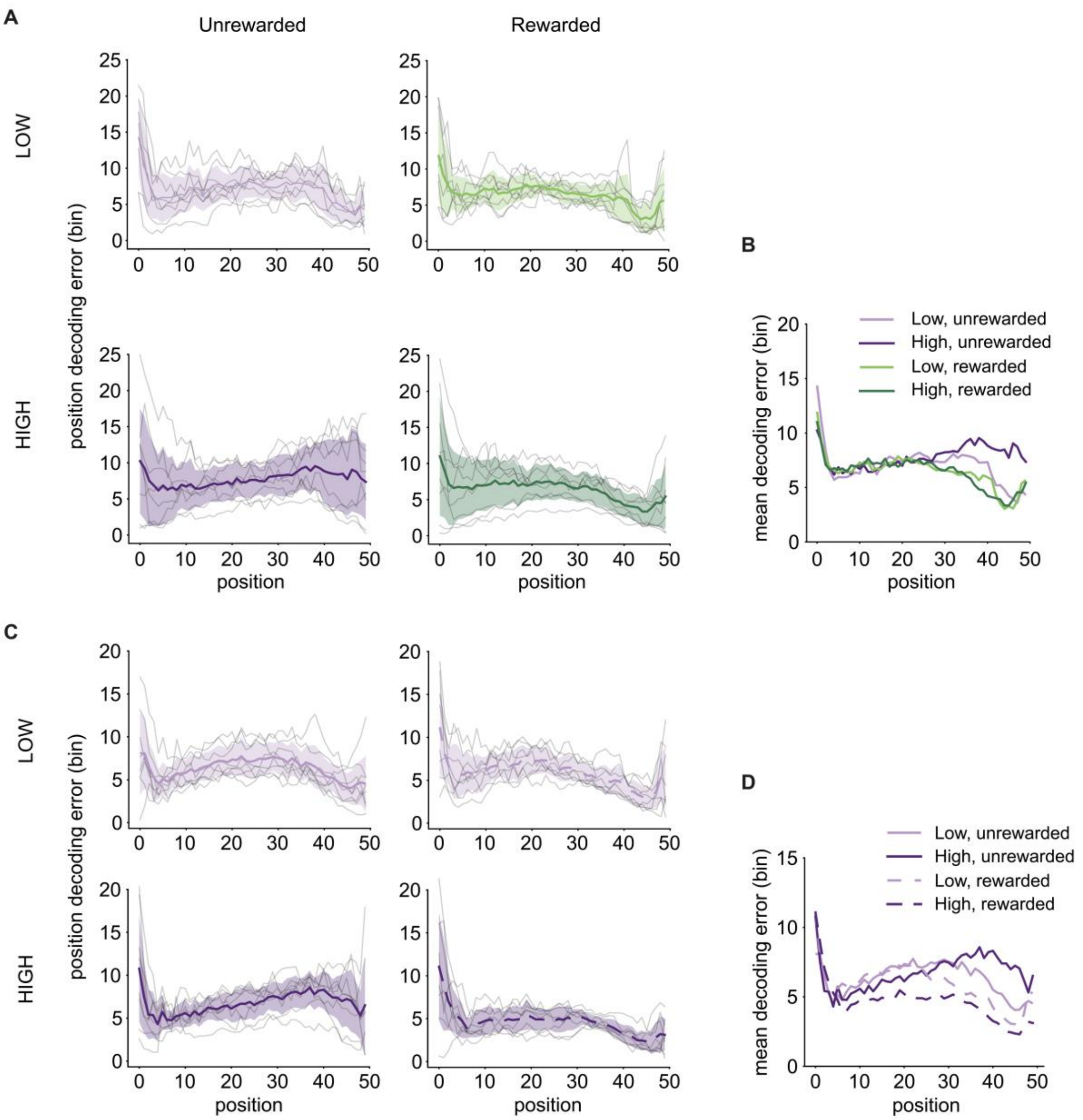

Fig. S6. Bayesian decoding of position in LOW vs. HIGH epochs.

(**A**) Cross-validated position decoding error in LOW (top) versus HIGH (bottom) epochs in the unrewarded (left) and in the rewarded (right) corridors as a function of position within the corridor in the Color condition. Grey lines indicate the mean (across laps) error of individual animals, colored lines and shading show mean ± SD across animals. Inset highlights that the mean decoding error around the reward zone diverges in the two corridors during learning: coding becomes more accurate near in the rewarded corridor whereas accuracy decreases in the unrewarded corridor.

(**B**) Mean decoding across animals. Note that the error around the reward zone (30-50) changes in the opposite direction in the two corridors during learning: coding becomes more accurate in the rewarded corridor whereas accuracy decreases in the unrewarded corridor.

(**C-D**) Same as **A-B** in the Pattern condition.

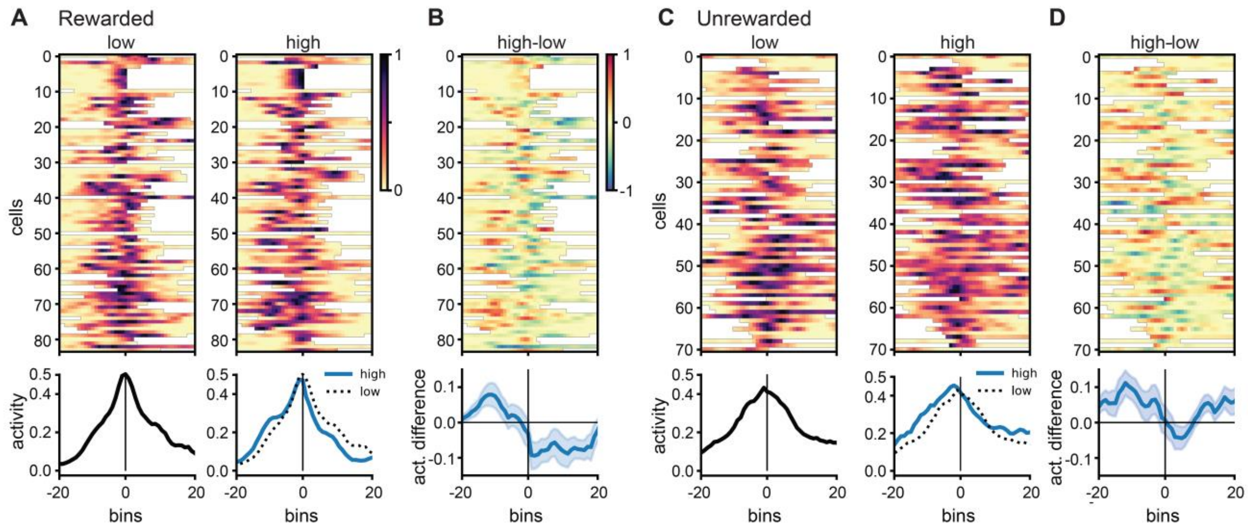

Fig. S7. Changes in single neuron tuning during learning.
(**A**) Left: Tuning curve (TC) of all neurons spatially tuned and selective for the rewarded corridor in the LOW epoch, aligned to the peak of their activity. Here we used TC estimated from the odd laps to align the TCs calculated from even laps. Note that some cells had their peak firing rate near the end of the corridor (white stripes: missing data). Bottom: TC averaged across all cells in the LOW epoch. Right: TC of the same neurons in the HIGH epoch, aligned to the peak in the low epoch for individual cells (top) and average across all cells (bottom).
(**B**) Difference between the TCs in the HIGH and in the LOW epochs for individual cells (top) and average across the population (bottom). Shading indicates standard error across the TCs with data at the given location.
(**C-D**) Same as **A-B** for the unrewarded corridor.

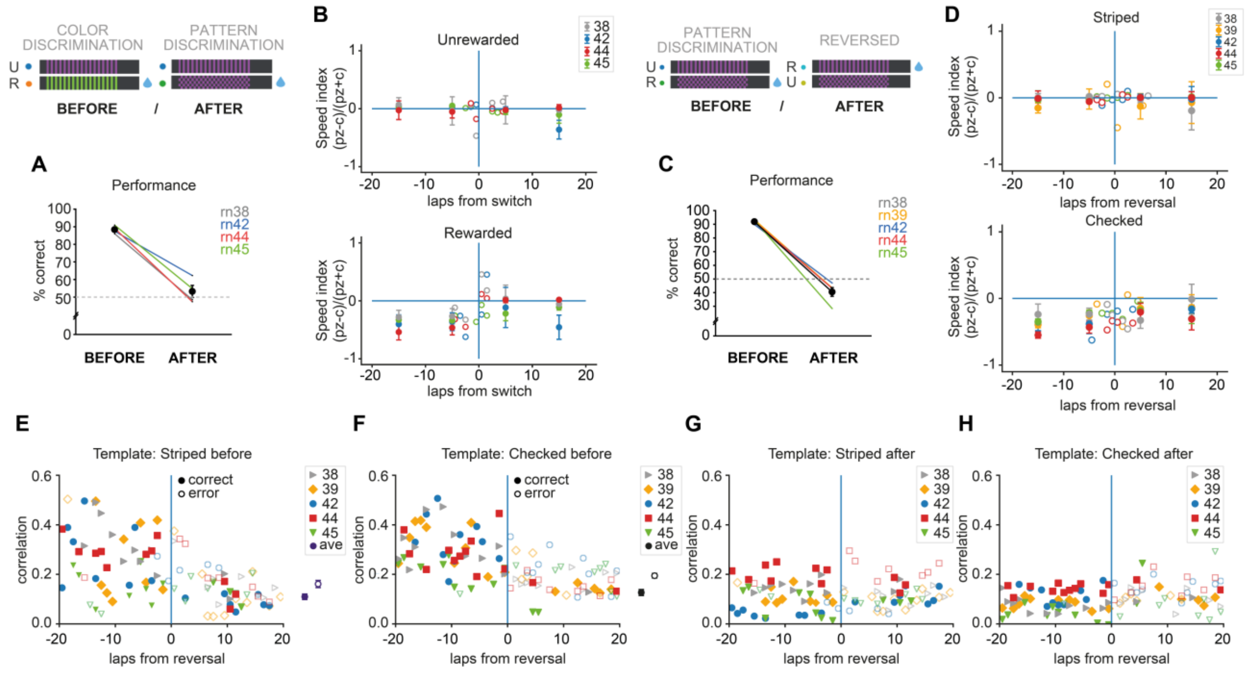

Fig. S8. Additional data related to task changes.

(**A**) Performance (% of correct laps) of the 4 mice (colors) before and after the Switch. Symbols and whiskers represent mean ± SE.

(**B**) Speed indices in the U and R corridors separately (averages of 10 laps (filled symbols) and the first and last two laps around the Switch (empty symbols)). In all 4 mice, the mean speed index in R in the first 2 laps after the switch increased above the 95th percentile of the speed index calculated from the 20 pre-switch laps.

(**C-D**) Same as **A-B** for the Reversal. Speed indices in **D** are shown in laps in the striped and checked corridors separately (averages of 10 laps and the first and last two laps around the Reversal).

(**E-H**) Lap-by-lap population activity correlations in 20 laps before and 20 laps after the Reversal to an average 'template' population activity. Templates were calculated before (**E-F**) or after (**G-H**) the analysed 40-lap window around the Reversal in individual corridors (checked or striped). Right subplots on **E-F,** average correlations calculated from post-swap correct and error laps separately. Mann-Whitney test: p=0.033 for striped corridor, p=0.005 for checked corridor.

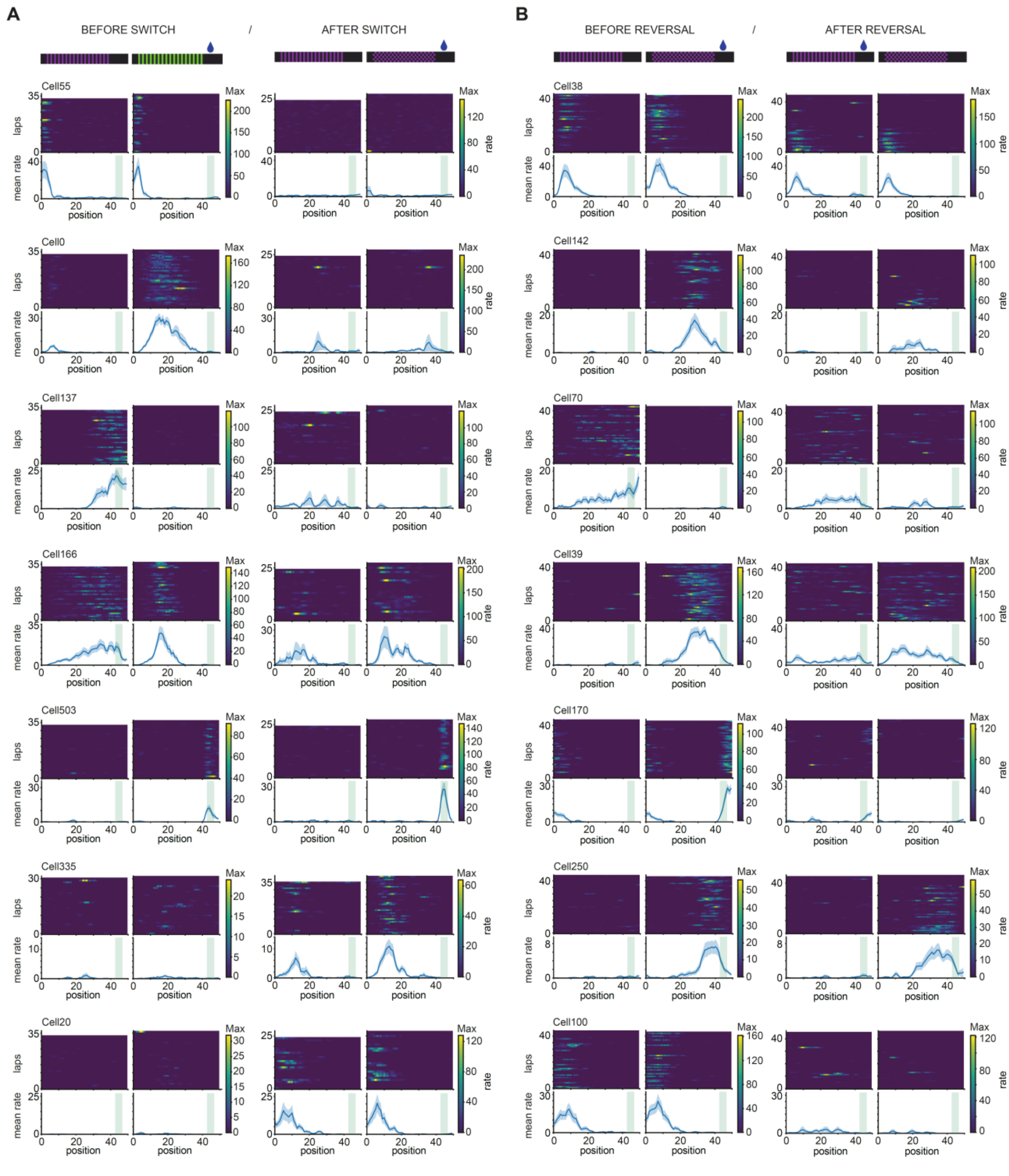

Fig. S9. Single neuron activity before and after task change.
Lap-by-lap activity and average estimated firing rate (mean ± SE, line and shading) per position bins along the U and R corridor for example neurons before (left) and after (right) switch (**A**) or reversal (**B**).

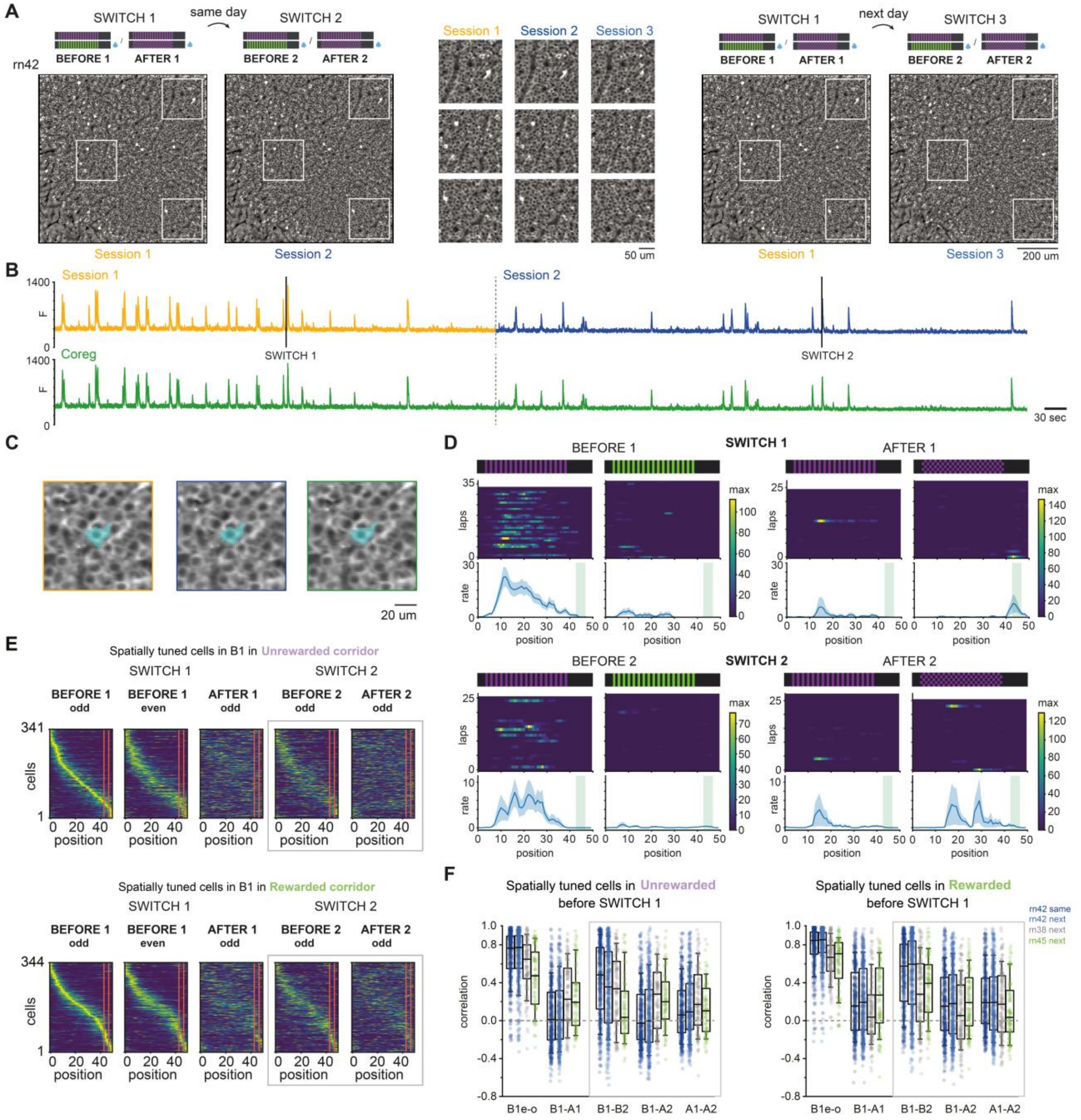

Fig. S10. Repeated switch in cross-registered CA1PNs

(**A**) Stability of the two-photon FOV across repeated switch sessions. Top, schematics of the experiment. Bottom, mean FOVs of images acquired during the indicated sessions recorded on the same day (left, Sessions 1-2) and one day apart (right, Sessions 1 and 3) in one example mouse (rn42). Middle: enlarged areas from the corresponding mean FOVs.

(**B**) Example Ca^{2+} traces used for the co-registration of the ROI shown in (**C**)**,** across imaging sessions (see *Methods*). Coreg, co-registration session.

(**C**) Example ROI (cyan, on mean enhanced image) recorded in Session 1 (yellow frame), Session 2 (blue frame) and the Co-registration session (green frame).

(**D**) Lap-by-lap Ca^{2+} activity rate maps (top) and tuning curves (bottom) of an example cell in Switch 1 and 2 in the Unrewarded (purple striped) and the Rewarded corridors (green striped before and purple squared after the switch).
(**E**) Normalized and sorted (by peak in B1, odd) Ca^{2+} activity rate maps of all cells spatially tuned before the first switch (B1; Switch 1) in the same session shown in **A-D.** Labels are as in **A.**
(**F**) Correlation between the tuning curves of the cells shown in (**E**) versus the tuning curve of the same cells in the same session (even vs. odd laps, B1e-o; dots: individual cells; box: 25-75 percentile; whiskers: 10 and 90 percentiles; horizontal line: median); before versus after the Switch 1 (B1-A1); before Switch 1 vs. before Switch 2/3 (B1-B2); before Switch 1 vs. after Switch 2/3 (B1-A2); and after Switch 1 vs. after Switch 2/3 (A1-A2). Color codes indicate different session pairs (n=3 mice). Cells typically retained their spatial tuning across different epochs in the same familiar condition (B1-B2) better than across switches (B1-A1 or B1-A2). Animal rn045 showed these effects only in the Rewarded corridor.

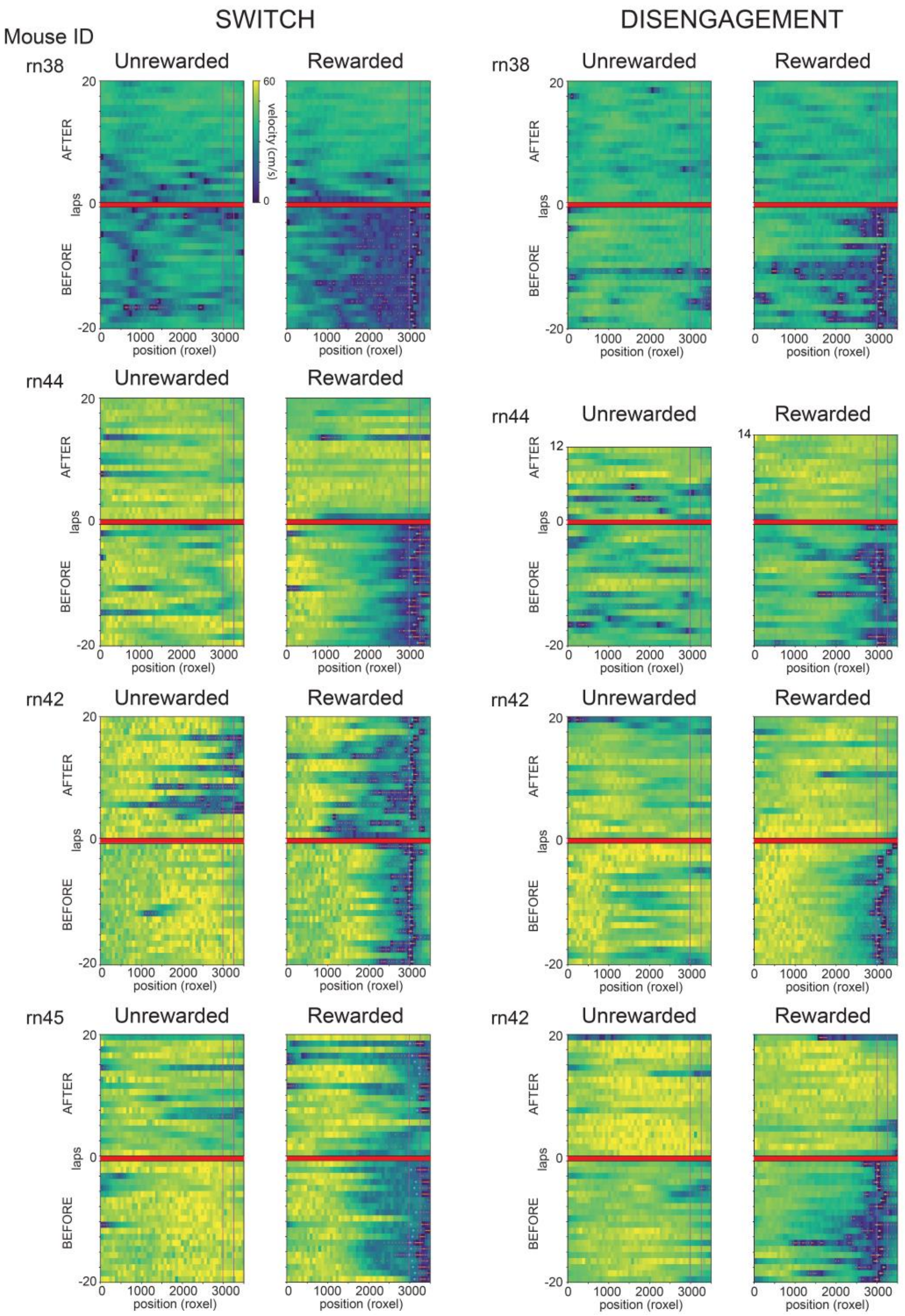

Fig. S11. Behaviors after switch vs. disengagement

Left, Velocity (color-coded) and licks (red dots) in 20 laps before and after the switch, in example switch sessions from four different mice. The horizontal bold red line indicates the time of the switch. Vertical thin red lines indicate the borders of the RZ. **Right**, Spontaneous behavioral changes without any condition change, identified as disengagement, in four sessions from three mice. The red lines indicate the time of the spontaneous behavioral change. Disengagement typically occurred after >400 laps run in the same condition.

Table S1. Statistical results (separate file)
Detailed results of all statistical analyses presented in Figs. 1, 3-7 and Fig. S4.
